# MetaInsight: An interactive web‐based tool for analyzing, interrogating, and visualizing network meta‐analyses using R‐shiny and netmeta

**DOI:** 10.1002/jrsm.1373

**Published:** 2019-10-11

**Authors:** Rhiannon K. Owen, Naomi Bradbury, Yiqiao Xin, Nicola Cooper, Alex Sutton

**Affiliations:** ^1^ NIHR Complex Reviews Support Unit, Department of Health Sciences, College of Life Sciences University of Leicester, George Davies Centre Leicester UK; ^2^ Zeeman Institute: Systems Biology and Infectious Disease Epidemiology Research (SBIDER), School of Life Sciences University of Warwick Coventry UK; ^3^ NIHR Complex Reviews Support Unit, Health Economics and Health Technology Assessment (HEHTA), Institute of Health and Wellbeing University of Glasgow Glasgow UK

## Abstract

**Background:**

Network meta‐analysis (NMA) is a powerful analysis method used to identify the best treatments for a condition and is used extensively by health care decision makers. Although software routines exist for conducting NMA, they require considerable statistical programming expertise to use, which limits the number of researchers able to conduct such analyses.

**Objectives:**

To develop a web‐based tool allowing users with only standard internet browser software to be able to conduct NMAs using an intuitive “point and click” interface and present the results using visual plots.

**Methods:**

Using the existing netmeta and Shiny packages for R to conduct the analyses, and to develop the user interface, we created the *MetaInsight* tool which is freely available to use via the web.

**Results:**

A package was created for conducting NMA which satisfied our objectives, and this is described, and its application demonstrated, using an illustrative example.

**Conclusions:**

We believe that many researchers will find our package helpful for facilitating NMA as well as allowing decision makers to scrutinize presented results visually and in real time. This will impact on the relevance of statistical analyses for health care decision making and sustainably increase capacity by empowering informed nonspecialists to be able to conduct more clinically relevant reviews. It is also hoped that others will be inspired to create similar tools for other advanced specialist analyses methods using the freely available technologies we have adopted.

**HIGHLIGHTS**

- A new tool that is freely available and conducts NMA via the web requiring no specialist software for the user to install but leveraging established analysis routines.
- The tool is interactive and uses an intuitive “point and click” interface, which can also carry out sensitivity analyses on existing NMAs in real time to help decision makers scrutinize the robustness of analysis findings.
- The tool was created using free, recently developed software, and we encourage others to generate similar tools using it for their areas of analysis expertise, particularly to increase the capacity of complex and specialist analysis approaches.
- It is hoped that this tool will increase the relevance of published meta‐analyses and, in the long term, contribute to improved health care decision making as a result.

## 1 INTRODUCTION

In this digital age where the quantity and complexity of data is rapidly expanding, and, in response, statistical analyses are increasing in complexity and becoming more specialist, there is a growing need for bespoke tools that facilitate the implementation and understanding of different analysis types. This is to ensure that researchers—other than statistical experts—are still fully engaged with state‐of‐the‐art approaches to data analysis.

In this paper, we describe and demonstrate one such tool—*MetaInsight*—we developed for network meta‐analysis (NMA), which we believe meets several unmet needs. This tool is web‐based, freely available (https://crsu.shinyapps.io/metainsightc), and runs on all modern internet browser applications. We encourage the reader to load and explore this tool (on a PC, tablet, or mobile phone) in conjunction with reading the remainder of this article.

The primary aim of this paper is to describe this tool and demonstrate its capabilities which include:

- Allowing the nonspecialist user to carry out a popular complex analysis method, NMA, in an interactive environment within an internet browser window;
- Providing an interactive environment in which an interested reader can further explore and scrutinize a published NMA by excluding studies on a case‐by‐case basis and, in doing so, check the robustness of the analysis findings; and
- Providing a platform by which the user can easily identify the impact of selecting different analysis methods through modifying the tools “point and click” options, since the tool gives immediate visual feedback regarding how changing options or data impacts the results.

A secondary goal is to promote the underlying ideas that drove the development of this specific tool and outline how the ideas were realized in the finished product. It is hoped that this will inspire and motivate others to develop similar resources as we believe that their availability will make a broader array of analyses approaches available to nonstatistical specialists and increase the relevance of published meta‐analyses for health care decision making. While much of the software utilized in developing the tool have only become available in recent years, all technologies used are freely available, and detailed knowledge of website development is not required to use them.

In the remainder of the paper, we briefly outline what NMA is and why it is an important methodology. Then, we explain why the tool is needed, underpinned by our experiences in developing, using, and teaching NMA methodology. We then provide a jargon‐free overview of the process and technologies used to develop the tool, followed by an outline of the scope of what the tool does and why it does it, illustrated with an example and figures taken directly from the tool. The code used to create the tool is also available in a technical specification document provided in Data S1 Supporting Information —this illuminates its capabilities and presents our vision of what accessible specialized data analysis tools should look like. A discussion, elaborating on important issues this work has raised, concludes the paper.

## 2 WHAT IS NETWORK META‐ANALYSIS?

**Figure 1:**
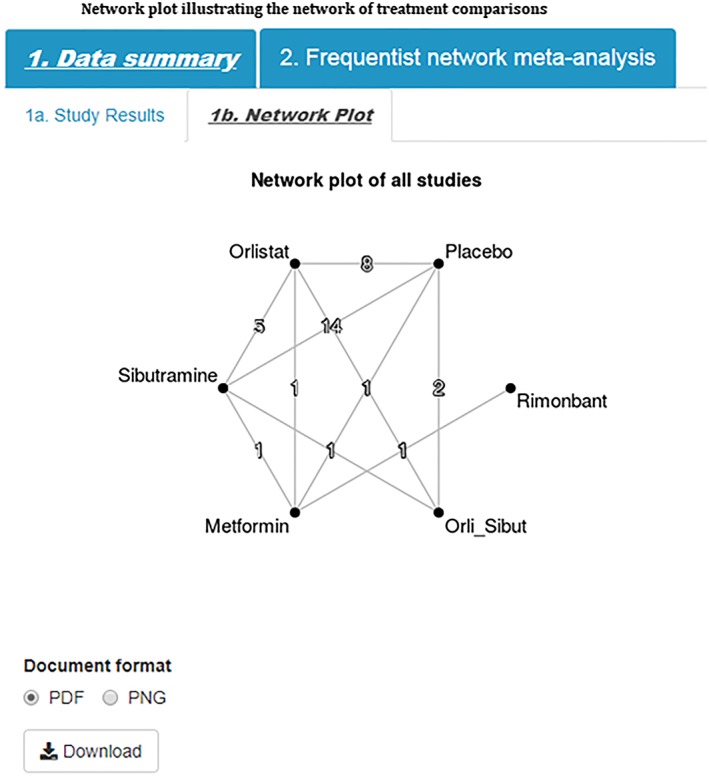
Network plot illustrating the network of treatment comparisons [Colour figure can be viewed at http://wileyonlinelibrary.com]

Meta‐analyses are established as a key component of the evaluation of medical interventions in terms of clinical effectiveness and cost effectiveness and as such are at the forefront of health care decision making.^1^ Pairwise meta‐analyses compare two interventions combining data from multiple head‐to‐head trials to obtain an overall summary effect size and its relative uncertainty. Network meta‐analysis (NMA) extends the pairwise approach with the aim to evaluate multiple interventions that may, or may not, have been directly compared in the trials.^2^, ^3^, ^4^ In doing so, this approach combines trials making different intervention comparisons, which together form a connected network of evidence (eg, Figure 1), to obtain relative treatment effects for all interventions compared with one another. In recent years, NMA methods have been widely adopted since they are able to answer the questions of clinical relevance such as, “which intervention is the ‘best’ overall?” Network meta‐analyses are regularly published in high‐quality medical journals, as well as being used in submissions to health technology assessment decision bodies such as NICE.

## 3 WHY IS THERE A NEED FOR THIS TOOL?

Currently, much of the software developed and used to implement NMA analyses requires knowledge of specialist statistical packages such as WinBUGS^5^ and R,^6^ and the output from such analyses can be extensive and complex. While it could be argued that, ideally, a statistician should always be involved in conducting a meta‐analysis, in reality, many groups who carry out systematic reviews containing meta‐analyses, including those within the Cochrane Collaboration, do not have the resources available for this to always be possible. Through teaching many NMA courses and supporting NIHR‐funded reviews, as part of the Complex Review Support Unit (CRSU), we have identified software as a barrier to greater adoption of the methods, and the need for results to be presented in more intuitive and user‐friendly ways to facilitate interpretation of results. This was the primary motivator for the development of the tool: to enable us to sustainably increase capacity by empowering informed nonspecialists to be able to conduct more clinically relevant reviews.

A further prior experience, which shaped the development of this tool, was through our research in collaboration with NICE, exploring how the health technology assessment decision‐making process could be improved. As a result of this collaboration, we built the Transparent interactive decision interrogator (TIDI), which allowed us to perform sensitivity analyses in real time in technology appraisal committees.^7^ Although a success, creation of the tool was complex, lengthy, and necessarily bespoke to a specific topic due to the technology limitations at the time. This tool motivated the development of *MetaInsight*, which focuses specifically on evidence syntheses with the advantage that *MetaInsight* is generalizable, allowing users to input their own data, and allows decision makers to perform sensitivity analyses in real time in technology appraisal meetings. A meta‐analysis should be seen as a lens through which to examine the current evidence rather than provide a totally objective “beyond criticism” result.^8^ We hope that *MetaInsight* allows users to explore this notion by easily changing basic NMA options, and excluding studies on a case‐by‐case basis to explore the robustness of results.

A final motivation was that, while we would consider ourselves to be experts in NMA methods, this does not mean that we would not appreciate a tool to facilitate the analysis ourselves(!) While packages such as R currently have tremendous support for the gamut of statistical methods developed, the user interfaces for such behemoths seem relatively neglected doing little to encourage wider uptake.

## 4 HOW THE TOOL WAS CREATED

We used existing technologies as much as possible to achieve our goals. Routines for conducting NMA in the statistical package R have reached a level of maturity and been peer reviewed.^9^ It seemed logical to utilize the frequentist versions of these as the analysis “heart” of the tool as implemented by the package netmeta. 10 A powerful package called Shiny 11 has been recently developed which enables web‐based user interfaces to be developed to interact with R, and this was used to provide the framework required for the tool. Although R is used as the “backbone” for the tool, it is accessed “behind the scenes” by the tool on the webserver so the user does not need to install it (or any other software beyond a web browser) on their device.

## 5 THE *METAINSIGHT* TOOL

The application can be accessed via the following URL https://crsu.shinyapps.io/metainsightc with full R code given in the web appendix.

The tool can interactively conduct NMA for both binary and continuous outcomes for fixed and random effects models^12^ and facilitates sensitivity analyses via the inclusion and exclusion of studies. Note that, since standard pairwise meta‐analysis can be seen as a special case of NMA, the tool can also be used to conduct standard meta‐analysis.

The tool contains a feature for the users to upload their own datasets into a webpage. Beyond this, operation of the tool is via a “point and click” interface via mouse/touchscreen. Graphical representations of the treatment network and various aspects of the results are provided. Any analyses/graphics produced can be saved in pdf format. The box illustrates typical usage of the app using an illustrative example dataset of pharmacological interventions for the treatment of obesity.^13^

## 6 ILLUSTRATIVE EXAMPLE OF THE USE OF THE *METAINSIGHT* TOOL APPLIED TO A MIXED TREATMENT COMPARISON OF PHARMACOLOGICAL INTERVENTIONS FOR THE TREATMENT OF OBESITY

The example dataset evaluated change from baseline in body mass index (BMI) for anti‐obesity interventions.^13^ There were six interventions of interest—placebo, orlistat, sibutramine, metformin, orlistat + sibutramine, and rimonbant—that were evaluated in various combinations across 24 studies. Twenty of these studies were two‐arm trials, three studies were three‐arm trials, and one study was a four‐arm trial. A network plot of all studies is given in (Figure 1). All studies reported estimates of mean difference from baseline in BMI, together with its corresponding uncertainty.

Step 1) Input data

**Figure 2:**
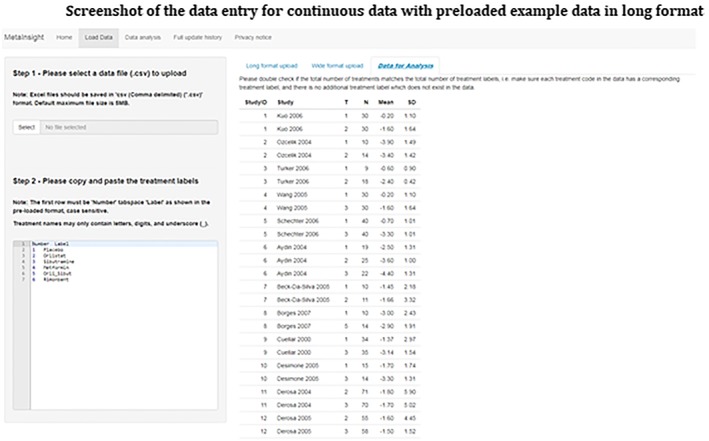
Screenshot of the data entry for continuous data with preloaded example data in long format [Colour figure can be viewed at http://wileyonlinelibrary.com]

The *MetaInsight* tool has a specific data entry page under the Load Data tab for each outcome type (continuous or binary)—both initially contain example data, together with their associated results, for illustration, didactic, and exploration purposes. Users can import their data easily by uploading a comma delimited (.csv) file in either long or wide format. In this example, we have continuous data (ie, outcome is change from baseline in BMI), and therefore we need to input data on study ID (as sequential and numerical code), study name, and treatment code (as sequential and numerical code), the number of participants, the mean treatment effects, and corresponding standard deviations for each arm of the study. Users may enter their own text label for each intervention by either copying‐and‐pasting from Microsoft Excel, or via any tab separated file. These text labels will appear in data and outputs of results. Users are reminded to keep labels short to allow for clear displays on visual outputs. An example of long format data entry is given in Figure 2.

Step 2) Choose NMA options data type and analysis

**Figure 3:**
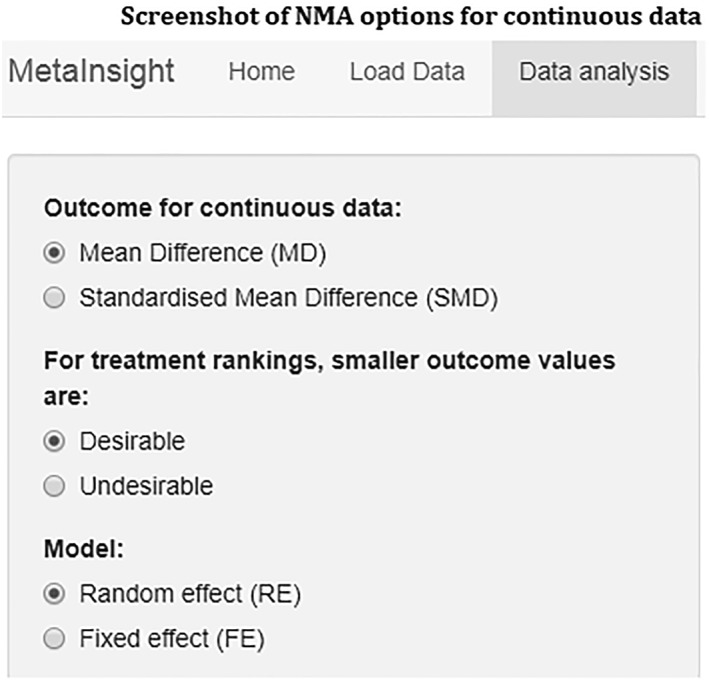
Screenshot of NMA options for continuous data

Having input your data, the next step is to select the outcome relevant for your data type under the Data Analysis tab. In this example, we have continuous data, and our outcome of interest is the mean difference in BMI. For the treatment rankings, the user must specify whether smaller outcome values are desirable or undesirable. In this example, as the outcome is change in BMI, smaller (more negative) values indicate a larger change in BMI and thus more effective treatments, and so we select desirable. Finally, the type of NMA model to fit to the data is selected—fixed or random effects^12^ (Figure 3).

Step 3) Visualize data and results

One of the key features of the application is the visualization of data and NMA results. The visual displays are divided in to two subtabs: Data summary and Frequentist NMA; each with their own subtabs including: Study results, Network plot, Forest plot, Comparison of all treatment pairs, and Inconsistency.

Step 4) Study Results

**Figure 4:**
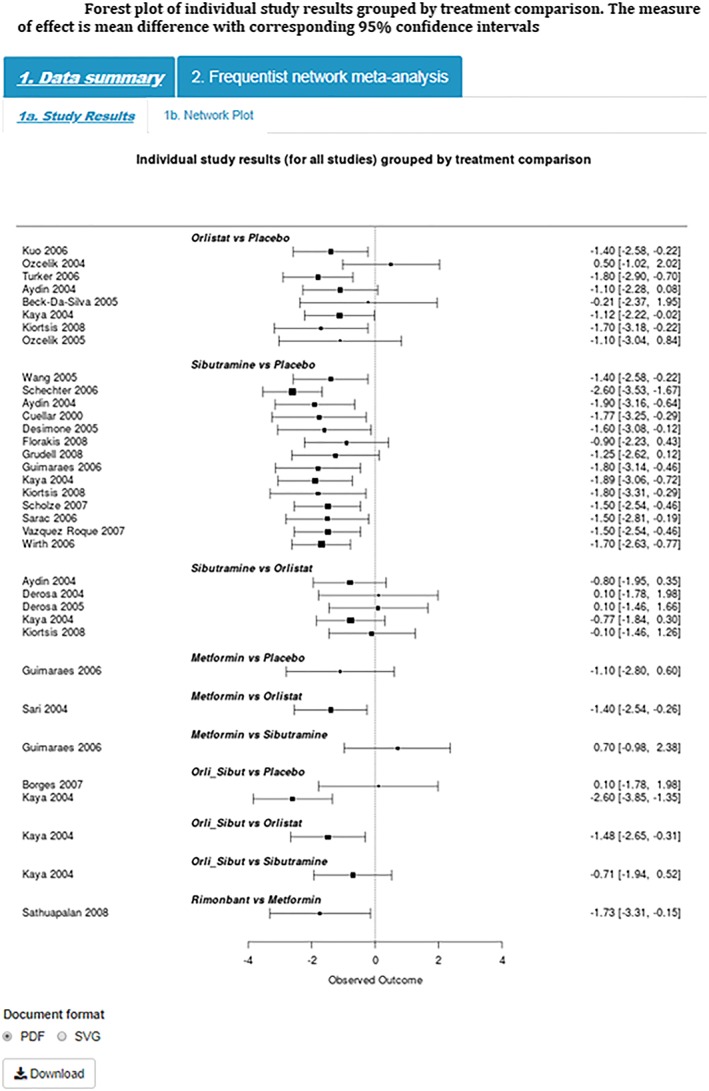
Forest plot of individual study results grouped by treatment comparison. The measure of effect is mean difference with corresponding 95% confidence intervals [Colour figure can be viewed at http://wileyonlinelibrary.com]

The most widely used plot in meta‐analysis is a forest plot which graphically displays the results from individual studies included in the meta‐analysis plotted on a common scale, in this example mean difference (Figure 4). This plot provides a visual display of the study specific data grouped by each pairwise treatment comparison allowing the user to visually examine the degree of heterogeneity between studies and identify any potentially outlying studies that the user may wish to examine further/exclude from the analysis as part of the sensitivity analysis (Step 4 below).

Step 5) Network Plot

Network plots provide a visual display of the network of evidence showing where interventions have been compared in head‐to‐head trials, and importantly whether there is a connected network of evidence, an essential requirement for NMA. The tool produces a network plot where each node on the plot represents an individual intervention with connecting lines between nodes indicating where one or more of the trials have evaluated both interventions on a head‐to‐head basis (Figure 1). The number of trials making each comparison is displayed on each line.^10^

Step 6) Forest Plot

**Figure 5:**
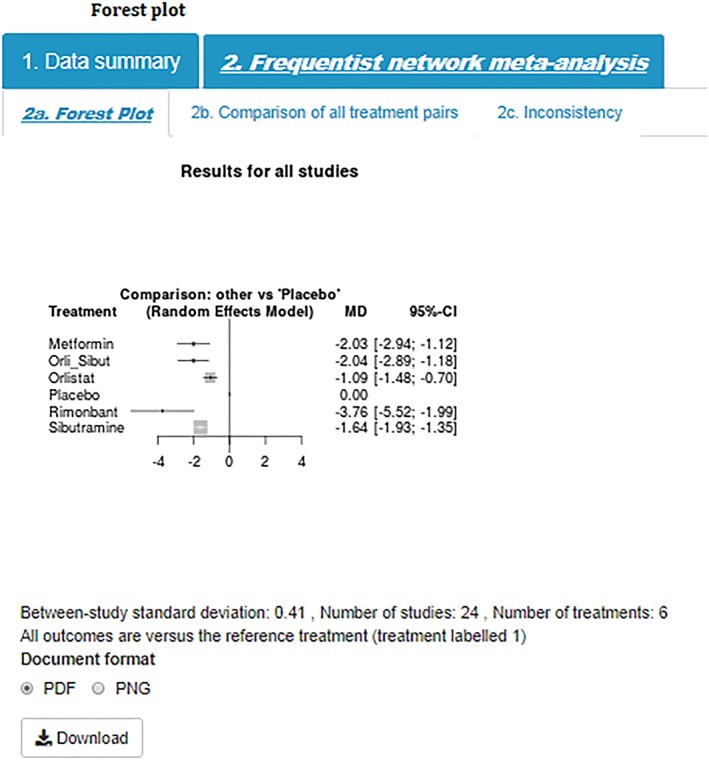
Forest plot [Colour figure can be viewed at http://wileyonlinelibrary.com]

In the interest of space and ease of interpretation, the tool uses a succinct and easily interpretable approach implemented within the netmeta package to display key results.^10^ These plots present the pooled effect estimates, and their associated uncertainty, for all interventions compared with the reference treatment (coded as treatment 1 in the data entry page—placebo in this example). The comparison plot also displays key information such as the between‐study standard deviation (indicating how much heterogeneity there is), the number of studies, and the number of treatments in the analysis. The results for this example are displayed in Figure 5 and show that rimonbant is likely to be the most effective treatment; that is, in relation to placebo (the reference treatment), rimonbant appeared to decrease BMI by approximately −3.76 kg/m^2^ (95%CI: −5.52, −1.99).

Step 7) Comparison of all treatment pairs

**Figure 6:**
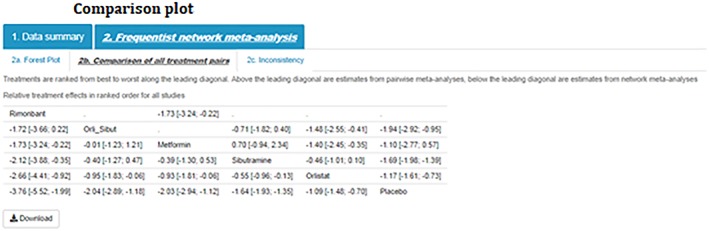
Comparison plot [Colour figure can be viewed at http://wileyonlinelibrary.com]

Comparison plots, or league tables, provide all pairwise comparisons in a NMA. Above the leading diagonal, in the upper triangle, are the treatment comparisons obtained from pairwise meta‐analyses. These are calculated as the treatment in the column versus the treatment in the row and presented as point estimates together with corresponding 95% confidence intervals. Below the leading diagonal, in the lower triangle, are the treatment comparisons obtained from the NMA. These are calculated as the treatment in the row versus the treatment in the column and presented as point estimates and corresponding 95% confidence intervals. The interventions are ordered from the most effective intervention to the least effective intervention along the leading diagonal (Figure 6).

Step 8) Inconsistency

**Figure 7:**
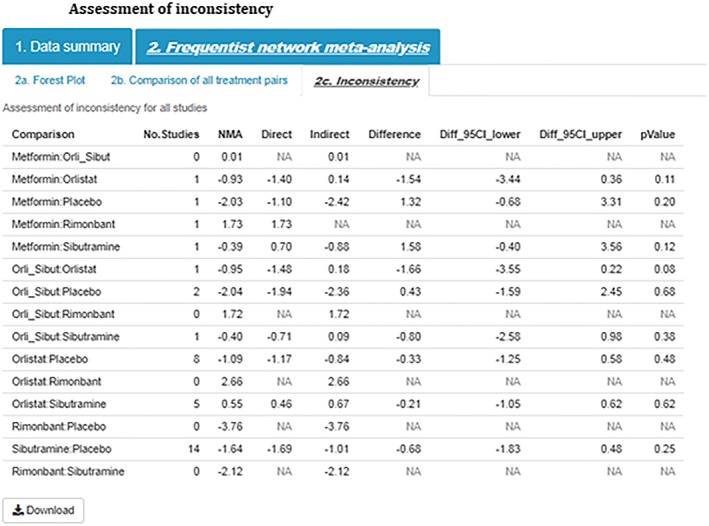
Assessment of inconsistency [Colour figure can be viewed at http://wileyonlinelibrary.com]

Consistencies between treatment effect estimates obtained from direct and indirect information are crucial for assumptions underpinning NMA to hold. Agreement between direct and indirect effect estimates can serve as a check for consistency of NMAs.^14^ The *MetaInsight* tool uses results obtained from the netmeta package^10^ to display the number of studies directly comparing treatments of interest, NMA treatment effect estimates, treatment effect estimates obtained from direct (ie, head to head) comparisons, and treatment effect estimates obtained from indirect information. For treatments that belong to a closed loop in the network of evidence (ie, there exists both direct and indirect information), the difference between the direct and indirect estimates is calculated together with the lower and upper limit of the 95% confidence interval. Differences between direct and indirect information are further quantified using *P* values where a low *P* value can be used to indicate conflicting evidence unlikely to be attributable to chance alone. In the example dataset (Figure 7), there does not appear to be any important estimated differences between direct and indirect information.

Step 9) Perform sensitivity analysis

**Figure 8:**
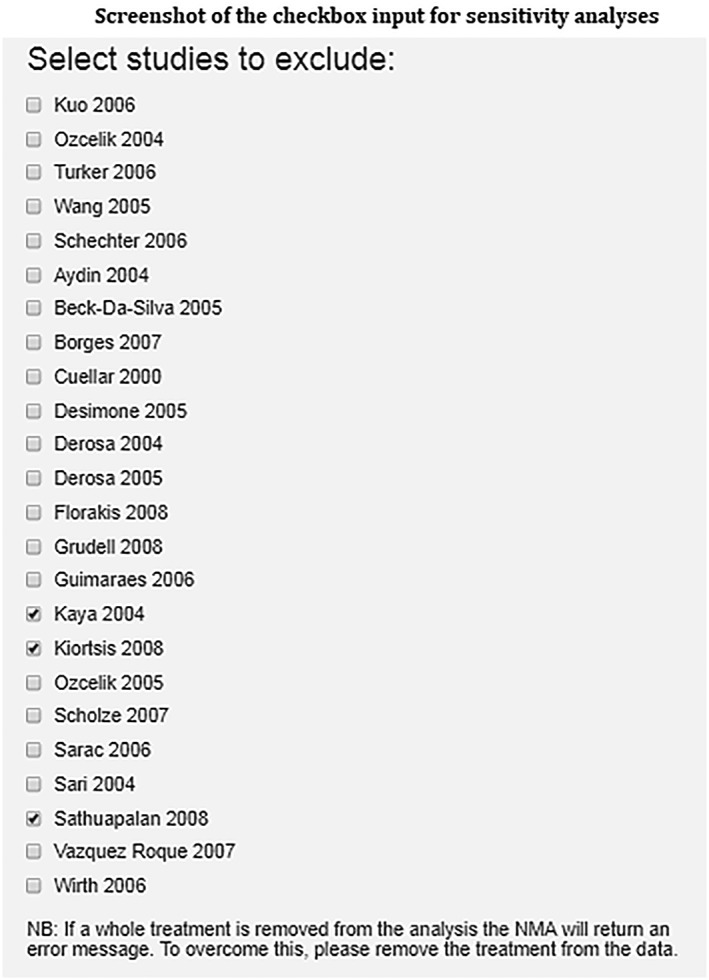
Screenshot of the checkbox input for sensitivity analyses

The influence of the different studies on the NMA results can be investigated through sensitivity analysis. For example, an analyst/clinician may want to explore the impact on the results of excluding a less rigorous, poorer quality study (ies) to assess the robustness of the analysis. As with all sensitivity analyses, there should be a clear rationale for including/excluding studies in a NMA and a description of included/excluded studies should be reported.^1^ Studies can easily be excluded from the analysis using a simple checkbox interface (Figure 8). The user can choose to exclude one or more studies, and the NMA will be updated and displayed alongside the complete case analysis which remains visible to facilitate comparison. For illustrative purposes only, in this particular example, we have chosen to exclude three studies (Kaya 2004, Kiortsis 2008, Sathuapalan 2008).

**Figure 9:**
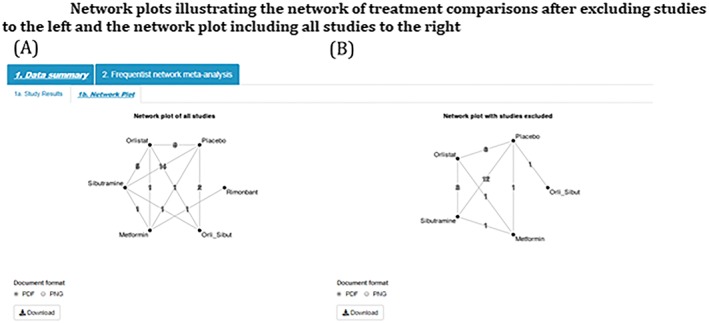
Network plots illustrating the network of treatment comparisons after excluding studies to the left and the network plot including all studies to the right [Colour figure can be viewed at http://wileyonlinelibrary.com]

The tool displays the network plot based on all studies entered into the tool (Figure 9A) next to the network excluding selected studies (Figure 9B). If, by excluding studies in the sensitivity analysis, an intervention is no longer connected to the network of evidence, eg, Rimonbant in Figure 9, then the disconnected intervention will disappear from the network plot. 1It is worth noting that whole treatments may be removed from the network and the analysis will still run providing that the remaining treatments are coded in sequential order. If a treatment is removed from the network, and the remaining treatments are no longer in sequential order, then the tool will return an error message and the analysis will not run.


**Figure 10:**
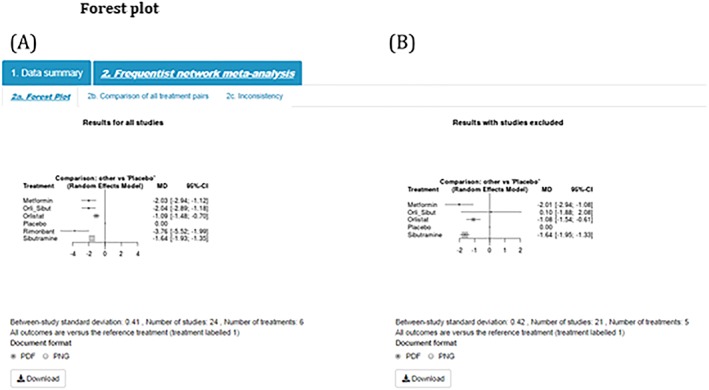
Forest plot [Colour figure can be viewed at http://wileyonlinelibrary.com]

**Figure 11:**
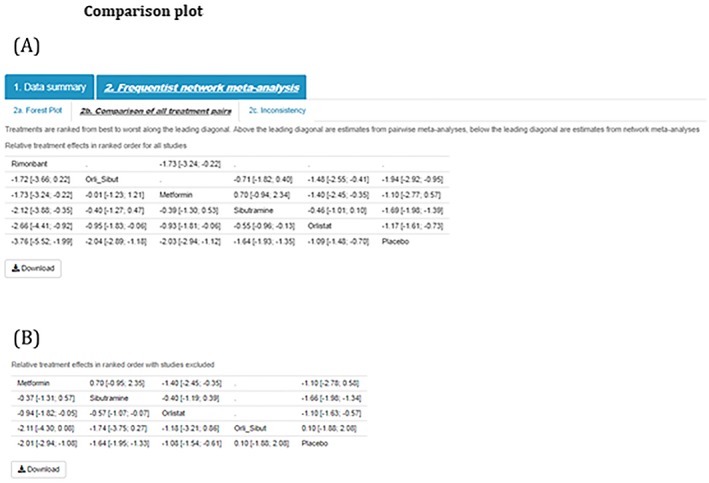
Comparison plot [Colour figure can be viewed at http://wileyonlinelibrary.com]

Similarly, summary forest plots (together with the number of studies included, number of treatments, and the estimated between‐study standard deviation) and comparison plots can be displayed for all studies (Figures 10A and 11A, respectively) and with selected studies excluded (Figures 10B and 11B, respectively).

Visual inspection of study results and assessment of inconsistency between direct and indirect evidence could also be evaluated after selecting studies to exclude whereby the plots will automatically update.

Step 10) Output results

The user has the option to download results as a portable document format (PDF), portable network graphic (PNG), or for study results, a scalar vector graphic (SVG). The ability to output results allows the user to include the produced figures directly in their NMA report.

## 7 DISCUSSION

This paper has presented a newly developed tool for NMA, which makes conducting this popular analysis available to a much wider audience than those who can use the existing code‐based approaches. In addition to researchers, we believe that the tool will be of interest to decision makers and students/educators as we have indicated throughout the paper.

*MetaInsight* allows both technical and nontechnical audiences to easily implement NMA and further explore data using user‐friendly interfaces. As with all statistical analyses, the ease of use of this tool may open the possibility of the noninformed user to misinterpret results. In this instance, we encourage the user to seek advice from technical experts. However, *MetaInsight* has been designed in such a way to limit human error, and consequently, the optional features implemented in *MetaInsight* are limited, such as fixed variables and variable names for data entry, numeric coding for treatments with the reference treatment in the network coded as 1, and restricting the number of arms in a study to a maximum of 6. A further limitation of the tool includes the inability to exclude studies based on a particular characteristic, eg, studies with a high risk of bias. Alternatively, the tool offers the ability to exclude studies on a case‐by‐case basis. For users wishing to fit more complex NMA models, including Bayesian NMA, and network meta‐regression models, for complex clinical scenarios, we encourage nontechnical users to consult with technical experts. Technical users may wish to make use of the full flexibility, modeling options, and output offered in the netmeta package^10^ as well as other packages such as metafor 15 in R, network 16 and network_graphs 17 in Stata, SAS, JAGS, WinBUGS, OpenBUGS, and Stan.^18^

The tool's framework leverages the power of the Shiny web interface for R, without which it would have been much more difficult for us to create. We believe the Shiny package has enormous untapped potential for use within medical research as it can connect all R analysis routines to the internet while circumventing the need to be fluent in the web development code languages HTM, CSS, and Javascript, as well as having web server programming proficiency.

Recently, others have also built web‐based applications for conducting systematic reviews, and we highlight the similarities and differences of these to *MetaInsight* below. Meta‐analysis via Shiny (MAVIS)^19^ conducts pairwise meta‐analyses, allowing users to input their own data and produce numerical and graphical outputs. Hence, this tool is similar in construction and functionality to ours, but is limited to only pairwise meta‐analysis and relies on more standard text‐based output formats. MetaBUS^20^ is an ambitious cloud‐based platform that semi‐automates the literature searching process by searching its own curated database of social science papers and associated results on a user‐requested topic and combines the findings in a pairwise meta‐analysis (also using R and Shiny “under the hood”). Hence, its major innovation is its ability to scrutinize a database and compile results data without the need for the user to do the preliminary stages of the analysis. Hence, while similar in spirit, both these apps have different scopes and remit to ours and neither can conduct NMA, our primary focus.

The Aggregate Data Drug Information System (ADDIS)^21^ and NetMetaXL^22^ share the aim of *MetaInsight* to increase capacity of network meta‐analyses for nontechnical users. ADDIS^21^ enables a semi‐automated construction of network meta‐analyses using a standalone “point and click” software package to perform Bayesian network meta‐analyses. NetMetaXL^22^ is based entirely within Microsoft Excel and provides a user‐friendly interface for users to perform Bayesian network meta‐analyses using WinBUGS. Both of these tools allow users to perform complex Bayesian network meta‐analyses and provide all outputs in a visually appealing way. *MetaInsight* offers a frequentist alternative using a user‐friendly interface based entirely on standard internet browsers and provides users with the opportunity to easily assess the robustness of results through the opportunity to perform sensitivity analyses using the “point and click” interactive interface. Another useful tool for facilitating the evaluation of NMA results is the freely available web‐based tool, CINeMA.^23^ CINeMA provides an interactive platform to assess the confidence in NMA results. Similarly to *MetaInsight*, CINeMA offers interactive network plots, league tables, and assessment of inconsistency. Further to this, CINeMA allows users to evaluate the contribution of individual studies on NMA estimates, assess heterogeneity in the network, and assess the overall risk of bias for contributing studies. Using packages such as CINeMA, we encourage users to explore the confidence in NMA results and contributions of individual studies, which could potentially inform sensitivity analyses using *MetaInsight*'s interactive platform.

There is much related further work possible in the creation of tools such as ours. Even within evidence synthesis, we have identified a need for, and started developing, a tool to conduct meta‐analysis of diagnostic test data. There are also many possible extensions to the existing tool, including the option to undertake analyses in a Bayesian framework, and the ability to include study‐level covariates.^24^ We are also looking to extend our previous work on web‐based visualization in three dimensions for network‐meta‐analysis^25^ (a coding expert programmed this app, but a similar tool would be possible with Shiny) to further develop the graphical outputs of this tool.

We are also exploring the possibility of being able to create and re‐publish the tool around a particular dataset that is “hard wired” into it. For example, when an NMA is published in a journal, our interactive front end to the associated dataset could be supplied as a “web extra” that could allow readers of the article the opportunity to explore the dataset and scrutinize the robustness of the analyses very quickly and easily. We think this is especially relevant for meta‐analysis as there are invariably subjective decisions made regarding the relevance of evidence in a systematic review, as well as end users wanting to ask subtle variations on the primary question of the review (eg, they may seek to explore a more targeted question relating to a subgroup of patients etc.).

## CONFLICT OF INTEREST

The author reported no conflict of interest.

## CONTRIBUTORS

R.K.O. is the guarantor for the article. All authors provided a substantial contribution to the design and implementation of the web tool, as well as writing sections of the manuscript, and approving the final version. R.K.O., N.B., and Y.X. developed the web tool.

## Supporting information

Data S1 Supporting Information

## Data Availability

Data sharing is not applicable to this article as no new data were created or analyzed in this study.
