# Supplementary material for: MetaInsight: An interactive web‐based tool for analyzing, interrogating, and visualizing network meta‐analyses using R‐shiny and netmeta
Source: Res Synth Methods. 2019 Oct 11;10(4):569–81. doi: 10.1002/jrsm.1373 (PMC6973101; doi:10.1002/jrsm.1373)
Supplement: Supplementary file 1 — Data S1 Supporting Information [file JRSM-10-569-s001.docx]

**Supplementary material**

R code for the user interface and server for MetaInsight (continuous) are described under ui.R and server.R, respectively.

**ui.R**

library(dplyr)

library(metafor)

library(netmeta)

library(shiny)

library(shinyAce)

library(rmarkdown)

library(knitr)

library(shinydashboard)

source("PlotFunctionsRKO.R", local = TRUE) # Plot functions

load("blank.rds") # Objects to store data for plot functions

shinyUI(navbarPage(id="meta",

"MetaInsight",

header = singleton(tags$head(includeScript("google_analytics2.js"))),

tabPanel(id="home", "Home",

h2("MetaInsight (continuous) V1.1**", tags$sup("Beta", style="color:#6CC0ED"), align= "left"),

h4(tags$a(href="https://crsu.shinyapps.io/metainsightb/", "For binary outcomes please click here.", target="_blank")),

fluidRow(

column(3, br(), br(),

img(src='network2.jpg', width=500, height=400, align = "center")

),

column(2),

column(5,

br(),

p(tags$strong("** New features updated on 15 March 2019 ** :")),

actionLink("history_click", "Click here to view a full update history of MetaInsight - continuous data"),

p(tags$ul(tags$li("This version now allows uploading data in 'long' format (1 study arm per row), in addition to the 'wide'

format (1 whole study per row).", tags$strong("For existing users (breaking change)"),": Please note that the ", tags$strong("required heading names have been changed"),

"slightly compared to the last version so you will need to change the names of the headings in your existing data files accordingly.

Please see instructions on the 'Load Data' page.")))

)

),

br(),

p("Rhiannon K Owen, Naomi Bradbury, Yiqiao Xin, Nicola Cooper, and Alex Sutton", align= "left"),

p("For feedback/questions about this app please contact", tags$a(href="mailto:rhiannon.owen@le.ac.uk", "rhiannon.owen@le.ac.uk"), align= "left"),

br(),

p("App powered by Rshiny. All frequentist satistical calculations are performed using netmeta package (Gerta RÃ¼cker, Guido Schwarzer, Ulrike Krahn and Jochem KÃ¶nig

2017). ",

tags$a(href="http://CRAN.R-project.org/package=netmeta", "netmeta: Network Meta-Analysis using Frequentist Methods.

R package version 0.9-8.",target="_blank")),

p("For users wishing to analyse large treatment networks or fit complex network meta-analysis models, please seek advice from technical experts."),

br(),

# adding the new div tag to the sidebar

p("THE SOFTWARE IS PROVIDED AS IS, WITHOUT WARRANTY OF ANY KIND, EXPRESS OR IMPLIED, INCLUDING BUT

NOT LIMITED TO THE WARRANTIES OF MERCHANTABILITY, FITNESS FOR A PARTICULAR PURPOSE AND NONINFRINGEMENT.

IN NO EVENT SHALL THE AUTHORS OR COPYRIGHT HOLDERS BE LIABLE FOR ANY CLAIM, DAMAGES OR OTHER LIABILITY,

WHETHER IN AN ACTION OF CONTRACT, TORT OR OTHERWISE, ARISING FROM, OUT OF OR IN CONNECTION WITH THE SOFTWARE

OR THE USE OR OTHER DEALINGS IN THE SOFTWARE."),

wellPanel(

fluidRow(

column(3, img(src='CRSUlogo.jpg', width=250, height=125)),

#img(src='CRSUlogo.jpg', width=250, height=165, align = "center"),

column(9, tags$div(class="header", checked=NA,

tags$p("Please click ", tags$a(href="http://www.nihrcrsu.org", "here ", target="_blank"), "for more information about the Complex Reviews Support Unit (CRSU)")

)

)

)

)

#img(src='CRSU.jpg', width=750, height=200, align = "center")

),

#########################

### Tab 2 - Load data ###

#########################

# Within the load data tab let users select a file to upload, the upload happens in a sidebarPanel on

# the left and the mainPanel will show the data once file uploaded. Code to show data is in the server

# section below

tabPanel("Load Data",

sidebarLayout(

sidebarPanel(

h4(tags$strong("Step 1 - Please select a data file (.csv) to upload")),

br(),

p(tags$strong("Note: Excel files should be saved in 'csv (Comma delimited) (*.csv)' format. Default maximum file size is 5MB.")),

fileInput(inputId="data", label="", buttonLabel="Select", placeholder="No file selected"),

br(),

tags$hr(),

#checkboxInput(inputId = "header", label = "First row as column headings", value = TRUE),

#checkboxInput(inputId = "stringAsFacSteptors", label = "stringAsfactors", FALSE),

#radioButtons(inputId = "sep", label="File Delimiter", choices=c(Comma=",", Semicolon=";", Tab="\t", Space= " "), selected=","),

h4(tags$strong("Step 2 - Please copy and paste the treatment labels")),

br(),

p(tags$strong("Note: The first row must be 'Number' tabspace 'Label' as shown in the pre-loaded format, case sensitive.")),

p(tags$strong(" Treatment names may only contain letters, digits, and underscore (_).")),

br(),

aceEditor("listCont2", value="Number\tLabel

1\tPlacebo

2\tOrlistat

3\tSibutramine

4\tMetformin

5\tOrli_Sibut

6\tRimonbant", mode="r", theme="eclipse")

),

mainPanel(

tabsetPanel(id="instructions",

tabPanel("Long format upload",

h2(tags$strong("Instructions for uploading long format data")),

br(),

p(tags$strong("MetaInsight allows data in either long format, or wide format. This tab provides instructions for long format data, where each row contains one treatment arm. Please follow Steps 1 and 2 to upload the data file and enter the treatment labels.

Instructions are as below.")),

h4(tags$strong("Step 1:")),

p(),

p("The long format data file should contain six columns. Headings of columns are case sensitive."),

p(tags$ul(tags$li("The", tags$strong("first"), "column should be labelled", tags$strong("StudyID"), "and contain the study identifier, starting from 1, then 2, 3, 4... etc."))),

p(tags$ul(tags$li("The", tags$strong("second"), "column should be labelled", tags$strong("Study"), "and contain the name (e.g., author,year) of the study. The study name must be unique for each study."))),

p(tags$ul(tags$li("The", tags$strong("third"), "column should be labelled", tags$strong("T"), "and contain the numerical treatment code used in each arm of the study.",

tags$strong("If applicable, your reference treatment (e.g. Placebo/Control)"), tags$strong(tags$u("needs to be labelled as 1."))))),

p(tags$ul(tags$li("The", tags$strong("fourth"), "column should be labelled", tags$strong("N"), "and contain the number of participants in each arm of the study."))),

p(tags$ul(tags$li("The", tags$strong("fifth"), "column should be labelled", tags$strong("Mean"), "and contain the mean value of the outcome in each arm of the study."))),

p(tags$ul(tags$li("The", tags$strong("sixth"), "column should be labelled", tags$strong("SD"), "and contain the standard deviation of the outcome in each arm of the study."))),

p("An example of this structure can be seen in the", tags$strong("'Data for Analysis'"), "tab."),

p("The csv file that is used to produce the example dataset can be downloaded from here:"),

# Button

downloadButton("downloadData", "Download the example dataset in long format"),

br(),

br(),

h4(tags$strong("Step 2:")),

p("Enter the labels to match with the numerical treatment codes in the data file. Labels should be short to allow for clear display on figures."),

p("Data can be copy and pasted from Excel or another tab separated file such as '.txt'"),

p("The default 'treatment labels' text file can be downloaded from here:"),

# Button

downloadButton("downloadlabel", "Download the example 'treatment labels' text file"),

br(),

p(),

p("This default dataset is from Gray, LJ. et al. A systematic review and mixed treatment comparison of pharmacological interventions for the treatment of obesity. Obesity reviews 13.6 (2012): 483-498."),

br(),

p(tags$strong("Note: The default dataset, pre-loaded on the 'Data for Analysis' tab, and its pre-loaded treatment labels will be used for analysis if no file is selected or no treatment labels are pasted. The 'Data for Analysis' tab will automatically update once a file is successfully loaded."))

),

tabPanel("Wide format upload",

h2(tags$strong("Instructions for uploading wide format data")),

br(),

p(tags$strong("MetaInsight allows data in either long format, or wide format. This tab provides instructions for wide format data, where each row contains all the treatment arms from one study. Please follow Steps 1 and 2 to upload the data file and enter the treatment labels.

Instructions are as below.")),

h4(tags$strong("Step 1:")),

p("Your data needs to have exactly the same variable names as in the example data which can be downloaded from here:"),

# Button

downloadButton("downloadDataWide", "Download the example dataset in wide format"),

br(),

p("Headings of columns are case sensitive."),

p(tags$ul(tags$li(tags$strong("StudyID"), "contains study identifier, starting from 1, then 2, 3, 4... etc."))),

p(tags$ul(tags$li(tags$strong("Study"), "contains name (e.g., author,year) of the study. The study name must be unique for each study."))),

p(tags$ul(tags$li(tags$strong("T.1, T.2, ..., up to T.6"), "contains treatment given for study arm 1, 2, ..., up to 6, respectively given as a numerical code"))),

p(tags$ul(tags$li(tags$strong("N.1, N.2, ..., up to N.6"), "contains number of participants in study arm 1, 2, ..., up to 6, respectively"))),

p(tags$ul(tags$li(tags$strong("Mean.1, Mean.2, ..., up to Mean.6"), "contains the mean value of the outcome in study arm 1, 2, ..., up to 6, respectively"))),

p(tags$ul(tags$li(tags$strong("SD.1, SD.2, ..., up to SD.6"), "contains standard deviation of the outcome in study arm 1, 2, ..., up to 6, respectively"))),

p(tags$strong("Note: If applicable, your reference treatment (e.g. Placebo/Control)",

tags$u("needs to be labelled as treatment 1"))),

p(tags$strong(" The maximum number of arms for each trial allowed in the MetaInsight app is 6.")),

br(),

h4(tags$strong("Step 2:")),

p("Enter the labels to match with the numerical treatment codes in the data file. Labels should be short to allow for clear display on figures."),

p("Data can be copy and pasted from Excel or another tab separated file such as '.txt'"),

p("The default 'treatment labels' text file can be downloaded from here:"),

# Button

downloadButton("downloadlabel2", "Download the example 'treatment labels' text file"),

br(),

p(),

p("This default dataset is from Gray, LJ. et al. A systematic review and mixed treatment

comparison of pharmacological interventions for the treatment of obesity. Obesity reviews 13.6 (2012): 483-498."),

br(),

p(tags$strong("Note: The default dataset, pre-loaded on the 'Data for Analysis' tab, and its pre-loaded treatment labels

will be used for analysis if no file is selected or no treatment labels are pasted. The 'Data for Analysis'

tab will automatically update once a file is successfully loaded."))

),

tabPanel("Data for Analysis",

p("Please double check if the total number of treatments matches the total number of treatment labels,

i.e. make sure each treatment code in the data has a corresponding treatment label,

and there is no additional treatment label which does not exist in the data."),

uiOutput("tb"))#,

#tabPanel("Default Data", tableOutput("defaultTable"))

)

)

)),

tabPanel("Data analysis",

sidebarLayout(

# Sidebar with a checkbox group input to select studies to exclude

sidebarPanel(

radioButtons("outcomeCont", "Outcome for continuous data:", c("Mean Difference (MD)" = "MD","Standardised Mean Difference (SMD)" = "SMD")),

radioButtons('rankoptsCont', 'For treatment rankings, smaller outcome values are:', c("Desirable" = "good", "Undesirable" = "bad")),

radioButtons("modelCont", "Model:", c("Random effect (RE)" = "RE", "Fixed effect (FE)" = "FE")),

h3("Select studies to exclude:"),

uiOutput("ChoicesCont2"), h5("NB: If a whole treatment is removed from the analysis the NMA will return an error message. To overcome this, please remove the treatment from the data."), width = 3

),

mainPanel(

tags$style(HTML("

.tabbable > .nav > li > a {background-color: white; color:#2196c4}

.tabbable > .nav > li > a[data-value='1. Data summary'] {background-color: #2196c4; color:white; font-size: 18px}

.tabbable > .nav > li > a[data-value='1a. Study Results'] {background-color: white;}

.tabbable > .nav > li > a[data-value='1b. Network Plot'] {background-color: white;}

.tabbable > .nav > li > a[data-value='2. Frequentist network meta-analysis'] {background-color: #2196c4; color:white; font-size: 18px}

.tabbable > .nav > li > a[data-value='2a. Forest Plot'] {background-color: white}

.tabbable > .nav > li > a[data-value='2b. Comparison of all treatment pairs'] {background-color: white;}

.tabbable > .nav > li > a[data-value='2c. Inconsistency'] {background-color: white;}

.tabbable > .nav > li[class=active] > a {font-weight:900;font-style: italic;text-decoration: underline }

")),

tabsetPanel(

tabPanel("1. Data summary", tabsetPanel(

tabPanel("1a. Study Results", plotOutput("forestPlotCont", height = "1000px", width = "800px"), radioButtons('format_freq0', 'Document format', c('PDF', 'SVG'), inline = TRUE), downloadButton('downloadStudyCont')),

tabPanel("1b. Network Plot",

column(6, plotOutput("netGraphStaticCont"), radioButtons('format_freq1', 'Document format', c('PDF', 'PNG'), inline = TRUE),

downloadButton('downloadNetworkCont')),

column(6, plotOutput("netGraphUpdatingCont"), radioButtons('format_freq2', 'Document format', c('PDF', 'PNG'), inline = TRUE),

downloadButton('downloadNetworkUpdateCont'))

)

)),

tabPanel("2. Frequentist network meta-analysis", tabsetPanel(

tabPanel("2a. Forest Plot",

column(6, plotOutput("ComparisonCont2", height = "400px", width = "400px"), textOutput("textcompcont"), textOutput("refcont4"), radioButtons('format_freq3', 'Document format', c('PDF', 'PNG'), inline = TRUE), downloadButton('downloadCompCont2')

),

column(6, plotOutput("SFPUpdatingContComp", height = "400px", width = "400px"), textOutput("textcont5"), textOutput("refcont3"), radioButtons('format_freq4', 'Document format', c('PDF', 'PNG'), inline = TRUE), downloadButton('downloadCompCont'))

),

tabPanel("2b. Comparison of all treatment pairs",

helpText("Treatments are ranked from best to worst along the leading diagonal. Above the leading diagonal are estimates from pairwise meta-analyses, below the leading diagonal are estimates from network meta-analyses"),

helpText("Relative treatment effects in ranked order for all studies"), tableOutput("rankChartStaticCont"), downloadButton('downloadRankCont', "Download"),

helpText("Relative treatment effects in ranked order with studies excluded"), tableOutput("rankChartUpdatingCont"), downloadButton('downloadRankUpdateCont')),

tabPanel("2c. Inconsistency",

helpText("Assessment of inconsistency for all studies"),

tableOutput("Incon1"), downloadButton('downloadInconCont', "Download"),

helpText("Assessment of inconsistency with studies excluded"),

tableOutput("Incon2"), downloadButton('downloadInconCont2', "Download")

)

)

)

)))),

tabPanel(id="history", "Full update history",

fluidRow(column(8,

p(tags$strong("** New features updated on 15 March 2019 ** :")),

p("Long and wide format data uploading:"),

p(tags$ul(tags$li("This version now allows uploading data in 'long' format (1 study arm per row), in addition to the 'wide'

format (1 whole study per row). The app will automatically detect the data format and prepare the data for

analysis accordingly. Instructions for uploading data are on the 'Load data' tab with subtabs for formatting

'long' and 'wide' data, respectively."))),

p(tags$ul(tags$li("Example datasets with associated treatment label files can now be downloaded which users can adapt to create

files of their data in the correct format for the app."))),

p(tags$ul(tags$li("Preparing the 'wide' format data is now more flexible: when there are less than 6 treatment arms, the users

just need to input the headings up to their maximum number of arms, i.e., you do not need to add the empty

columns with headings in the data anymore."))),

p(tags$ul(tags$li(tags$strong("For existing users (breaking change)"),": Please note that the ", tags$strong("required heading names have been changed"),

"slightly compared to the last version so you will need to change the names of the headings in your existing data files accordingly.

Please see instructions on the 'Load data' page.")))

))

)

)

)

**server.R**

library(dplyr)

library(metafor)

library(netmeta)

library(shiny)

library(shinyAce)

library(rmarkdown)

library(knitr)

source("PlotFunctionsRKO.R", local = TRUE) # Plot functions

load("blank.rds") # Objects to store data for plot functions

shinyServer(function(input, output, session) {

showModal(modalDialog(

title = "Important message",

easyClose = FALSE,

p(tags$strong("In accordance with Data Protection legislation, we would like to inform you of the following before you use our website:

"), "We collect your usage data within the MetaInsight app to perform analytics and improve our app. By clicking",

tags$i(tags$u("I Agree")), "below, you consent to the use of data by us through Google Analytics.

For details of policy, please check",tags$a(href="https://policies.google.com/privacy?hl=en", "Google Privacy & Terms.",target="_blank") ),

br(),

modalButton("I Agree"),

footer = NULL

))

### view the full update history

observeEvent(input$history_click, {

newvalue <- "history"

updateNavbarPage(session,"meta", selected="Full update history")

}

#############################################

############ Load Data ################

#############################################

auditC <- read.csv("./TRY.csv")

#auditC <- read.csv("C:/Users/yx8w/Desktop/TRY.csv")

#####

# Default data

#####

defaultData <- reactive({

auditC

})

############################################

#####Downloadable csv and labels of example dataset

############################################

datad <- as.data.frame(auditC)

##### in the 'upload long data' tab

output$downloadData <- downloadHandler(

filename <- function() {

paste("MetaInsightdataLONG","csv", sep = ".")

},

content <- function(file){

file.copy("./TRY.csv", file)

}

)

output$downloadlabel <- downloadHandler(

filename <- function() {

paste("treatmentlabels","txt", sep = ".")

},

content <- function(file){

file.copy("./defaultlabels.txt", file)

}

)

##### in the 'UPload wide data' tab

output$downloadDataWide <- downloadHandler(

filename <- function() {

paste("MetaInsightdataWIDE","csv", sep = ".")

},

content <- function(file){

file.copy("./TRY_WIDE.csv", file)

}

)

output$downloadlabel2 <- downloadHandler(

filename <- function() {

paste("treatmentlabels","txt", sep = ".")

},

content <- function(file){

file.copy("./defaultlabels.txt", file)

}

)

#####

# Make data reactive

#####

data <- reactive({

file1 <- input$data

if(is.null(file1)){return(auditC)}

else

a <- read.table(file = file1$datapath, sep =",", header=TRUE, stringsAsFactors = FALSE)

})

#####

# Data analysis tab

#####

# Create a table which displays the raw data just uploaded by the user

output$rawtable <- renderTable({

if(is.null(data())){return()}

data()

})

# In the "Load data" tab (created in the UI section) we divide the main panel into multiple tabs and add the content

# When there is no data loaded display the instructions for how the data should be formatted

# Once data is oaded display the raw data

output$tb <- renderUI({

if(is.null(data())){return("Please select a file to upload.")}

else

tableOutput("rawtable")

})

####################################################################################

#########

### Get studies for check box input

#########

output$ChoicesCont2 <- renderUI({

newData <- data()

newData1 <- as.data.frame(newData)

# if the data in long format, then the dataset will contain exactly six columns.

# whereas the wide format will contain at least 2+4*2=10 columns.

if (ncol(newData1)==6){

newData2<-newData1[order(newData1$StudyID, -newData1$T), ]

# create counting variable for number of arms within each study.

newData2$number<- ave(as.numeric(newData2$StudyID),newData2$StudyID,FUN=seq_along)

# reshape

data_wide <- reshape(newData2, timevar = "number",idvar = c("Study", "StudyID"), direction = "wide")

}

else {

data_wide<- newData1

}

checkboxGroupInput("exclusionCont2",

label = NULL,

choices = as.character(data_wide$Study))

})

######

# Grouped Forest Plot (all studies) - Continuous Data

######

make_netStudyCont = function() {

newData <- data()

newData1 <- as.data.frame(newData)

if (ncol(newData1)==6){

newData2<-newData1[order(newData1$StudyID, -newData1$T), ]

}

else {

# if the data is in wide form, the following code order the treatment in a descending order

data_wide<- newData1

numbertreat=(ncol(newData1)-2)/4

# generate additional columns if less than 6 arms for the reshape

if (numbertreat < 6) {

for (k in (numbertreat+1):6) {

data_wide[c(paste0("T.",k),paste0("N.",k),paste0("Mean.",k),paste0("SD.",k))]<-NA

}

}

widetolong <- reshape(data_wide, direction = "long",

varying = 3:ncol(data_wide),

times=c(".1", ".2", ".3", ".4", ".5", ".6"), sep=".", idvar= c("StudyID", "Study"))

widetolong<-subset(widetolong, select=-time)

newData2 <- widetolong[!is.na(widetolong$T), ]

newData2<-newData2[order(newData2$StudyID, -newData2$T), ]

}

# create counting variable for number of arms within each study.

newData2$number<- ave(as.numeric(newData2$StudyID),newData2$StudyID,FUN=seq_along)

# reshape

data_wide <- reshape(newData2, timevar = "number",idvar = c("Study", "StudyID"), direction = "wide")

numbertreat=max(newData2$number)

# generate additional columns if less than 6 arms.

if (numbertreat < 6) {

for (k in (numbertreat+1):6) {

data_wide[c(paste0("T.",k),paste0("N.",k),paste0("Mean.",k),paste0("SD.",k))]<-NA

}

}

# Get subset of data to use

data_sub <- filter(data_wide, !Study %in% input$exclusionCont2)

if (input$outcomeCont=="MD"){

d1<- pairwise(treat=list(T.1,T.2,T.3,T.4,T.5,T.6),n=list(N.1,N.2,N.3,N.4,N.5,N.6),mean=list(Mean.1,Mean.2,Mean.3,Mean.4,Mean.5,Mean.6),sd=list(SD.1,SD.2,SD.3,SD.4,SD.5,SD.6),data=data_sub,sm="MD")

}

else{

d1<- pairwise(treat=list(T.1,T.2,T.3,T.4,T.5,T.6),n=list(N.1,N.2,N.3,N.4,N.5,N.6),mean=list(Mean.1,Mean.2,Mean.3,Mean.4,Mean.5,Mean.6),sd=list(SD.1,SD.2,SD.3,SD.4,SD.5,SD.6),data=data_sub,sm="SMD")

}

data_final<- merge(d1,data_wide,by="StudyID")

treat_list <- read.csv(text=input$listCont2, sep = "\t")

lstx <- treat_list$Label

ntx <- length(lstx)

text_label <- character()

n_stud <- integer()

for (i in 1:ntx) {

for (j in 1:ntx) {

if (nrow(d1[(d1$treat1 == i & d1$treat2 == j),]) > 0) {

text_label <- c(paste(lstx[i], "vs", lstx[j]), text_label)

n_stud <- c(n_stud, nrow(d1[(d1$treat1 == i & d1$treat2 == j),]))

}

}

}

gaps <- integer(length(n_stud))

n_stud <- rev(n_stud)

for (k in 1:length(n_stud)) {

if (k == 1) {

gaps[k] <- n_stud[k] + 1

}

else {

gaps[k] <- gaps[k-1] + n_stud[k] + 2

}

}

lines <- rev(c(1:(nrow(d1) + 2*length(text_label)-1)))

lines <- lines[!lines %in% gaps]

lines <- lines[!lines %in% (gaps+1)]

forest(d1$TE, d1$seTE, slab = paste(data_final$Study.y), subset = order(d1$treat1, d1$treat2), ylim = c(1, nrow(d1) + 2*length(text_label) + 2), rows=lines)

text(-6, gaps, pos=4, font = 4, text_label)

title("Individual study results (for all studies) grouped by treatment comparison")

}

output$forestPlotCont <- renderPlot({

withProgress(message = 'Loading', value = 0, {

# Number of times we'll go through the loop

n <- 10

for (i in 1:n) {

# Increment the progress bar, and update the detail text.

incProgress(1/n, detail = paste(""))

}

})

make_netStudyCont()

})

output$downloadStudyCont <- downloadHandler(

filename = function() {

paste0('StudyResults.', input$format_freq0)

},

content = function(file) {

if (input$format_freq0=="PDF"){pdf(file=file)}

else {svg(file=file)}

make_netStudyCont()

dev.off()

}

)

##########

### Network Plot static - continuous

##########

make_netgraphCont = function() {

#### same code for inputting the data

newData <- data()

newData1 <- as.data.frame(newData)

if (ncol(newData1)==6){

newData2<-newData1[order(newData1$StudyID, -newData1$T), ]

# create counting variable for number of arms within each study.

newData2$number<- ave(as.numeric(newData2$StudyID),newData2$StudyID,FUN=seq_along)

# reshape

data_wide <- reshape(newData2, timevar = "number",idvar = c("Study", "StudyID"), direction = "wide")

numbertreat=max(newData2$number)

}

else {

data_wide<- newData1

numbertreat=(ncol(newData1)-2)/4

}

# generate additional columns if less than 6 arms.

if (numbertreat < 6) {

for (k in (numbertreat+1):6) {

data_wide[c(paste0("T.",k),paste0("N.",k),paste0("Mean.",k),paste0("SD.",k))]<-NA

}

}

#####

d1<- pairwise(treat=list(T.1,T.2,T.3,T.4,T.5,T.6),n=list(N.1,N.2,N.3,N.4,N.5,N.6),mean=list(Mean.1,Mean.2,Mean.3,Mean.4,Mean.5,Mean.6),sd=list(SD.1,SD.2,SD.3,SD.4,SD.5,SD.6),data=data_wide,sm="MD")

treat_list <- read.csv(text=input$listCont2, sep = "\t")

lstx <- treat_list$Label

ntx <- length(lstx)

# Static NMA of all studies

netStatic <- netmeta(TE, seTE, treat1, treat2, studlab, data = d1, subset=NULL,

sm = "MD", level=0.95, level.comb=0.95,

comb.random=TRUE, reference.group="",

all.treatments=NULL, seq=NULL, tau.preset=NULL,

tol.multiarm = 0.05, warn=TRUE)

netgraph(netStatic, lwd=2, number.of.studies = TRUE, plastic=FALSE, points=TRUE, labels= lstx, cex=1.25, cex.points=2, col.points=1, col=8)

title("Network plot of all studies")

}

output$netGraphStaticCont <- renderPlot ({

make_netgraphCont()

}, height="auto", width = "auto")

output$downloadNetworkCont <- downloadHandler(

filename = function() {

paste0('Network.', input$format_freq1)

},

content = function(file) {

if (input$format_freq1=="PDF"){pdf(file=file)}

else {png(file=file)}

make_netgraphCont()

dev.off()

}

)

##########

### Network Plot updating

##########

make_netgraphUpdateCont = function(){

#### same code for inputting the data

newData <- data()

newData1 <- as.data.frame(newData)

if (ncol(newData1)==6){

newData2<-newData1[order(newData1$StudyID, -newData1$T), ]

# create counting variable for number of arms within each study.

newData2$number<- ave(as.numeric(newData2$StudyID),newData2$StudyID,FUN=seq_along)

# reshape

data_wide <- reshape(newData2, timevar = "number",idvar = c("Study", "StudyID"), direction = "wide")

numbertreat=max(newData2$number)

}

else {

data_wide<- newData1

numbertreat=(ncol(newData1)-2)/4

}

# generate additional columns if less than 6 arms.

if (numbertreat < 6) {

for (k in (numbertreat+1):6) {

data_wide[c(paste0("T.",k),paste0("N.",k),paste0("Mean.",k),paste0("SD.",k))]<-NA

}

}

#####

treat_list <- read.csv(text=input$listCont2, sep = "\t")

lstx <- treat_list$Label

ntx <- length(lstx)

# Get subset of data to use

data_sub <- filter(data_wide, !Study %in% input$exclusionCont2)

d1_sub <- d1<- pairwise(treat=list(T.1,T.2,T.3,T.4,T.5,T.6),n=list(N.1,N.2,N.3,N.4,N.5,N.6),mean=list(Mean.1,Mean.2,Mean.3,Mean.4,Mean.5,Mean.6),sd=list(SD.1,SD.2,SD.3,SD.4,SD.5,SD.6),data=data_sub,sm="MD")

# Network meta-analysis

net1 <- netmeta(TE, seTE, treat1, treat2, studlab, data = d1_sub, subset=NULL,

sm = "MD", level=0.95, level.comb=0.95,

comb.random=TRUE, reference.group="",

all.treatments=NULL, seq=NULL, tau.preset=NULL,

tol.multiarm = 0.05, warn=TRUE)

netgraph(net1, lwd=2, number.of.studies = TRUE, plastic=FALSE, points=TRUE, labels= lstx, cex=1.25, cex.points=2, col.points=1, col=8)

title("Network plot with studies excluded")

}

output$netGraphUpdatingCont <- renderPlot({

withProgress(message = 'Loading', value = 0, {

# Number of times we'll go through the loop

n <- 10

for (i in 1:n) {

# Increment the progress bar, and update the detail text.

incProgress(1/n, detail = paste(""))

}

})

make_netgraphUpdateCont()

})

output$downloadNetworkUpdateCont <- downloadHandler(

filename = function() {

paste0('NetworkUpdate.', input$format_freq2)

},

content = function(file) {

if (input$format_freq2=="PDF"){pdf(file=file)}

else {png(file=file)}

make_netgraphUpdateCont()

dev.off()

}

)

##########

### Comparison and rank table - Continuous

##########

make_netrankCont = function() {

#### same code for inputting the data

newData <- data()

newData1 <- as.data.frame(newData)

if (ncol(newData1)==6){

newData2<-newData1[order(newData1$StudyID, -newData1$T), ]

# create counting variable for number of arms within each study.

newData2$number<- ave(as.numeric(newData2$StudyID),newData2$StudyID,FUN=seq_along)

# reshape

data_wide <- reshape(newData2, timevar = "number",idvar = c("Study", "StudyID"), direction = "wide")

numbertreat=max(newData2$number)

}

else {

data_wide<- newData1

numbertreat=(ncol(newData1)-2)/4

}

# generate additional columns if less than 6 arms.

if (numbertreat < 6) {

for (k in (numbertreat+1):6) {

data_wide[c(paste0("T.",k),paste0("N.",k),paste0("Mean.",k),paste0("SD.",k))]<-NA

}

}

#####

if (input$outcomeCont=="MD"){

d1<- pairwise(treat=list(T.1,T.2,T.3,T.4,T.5,T.6),n=list(N.1,N.2,N.3,N.4,N.5,N.6),mean=list(Mean.1,Mean.2,Mean.3,Mean.4,Mean.5,Mean.6),sd=list(SD.1,SD.2,SD.3,SD.4,SD.5,SD.6),data=data_wide,sm="MD")

}

else{

d1<- pairwise(treat=list(T.1,T.2,T.3,T.4,T.5,T.6),n=list(N.1,N.2,N.3,N.4,N.5,N.6),mean=list(Mean.1,Mean.2,Mean.3,Mean.4,Mean.5,Mean.6),sd=list(SD.1,SD.2,SD.3,SD.4,SD.5,SD.6),data=data_wide,sm="SMD")

}

treat_list <- read.csv(text=input$listCont2, sep = "\t")

lstx <- treat_list$Label

ntx <- length(lstx)

d1$treat1 <- factor(d1$treat1,

levels = c(1:ntx),

labels = as.character(treat_list$Label))

d1$treat2 <- factor(d1$treat2,

levels = c(1:ntx),

labels = as.character(treat_list$Label))

# Static NMA of all studies

if (input$modelCont=="RE" & input$outcomeCont=="MD"){

netStatic <- netmeta(TE, seTE, treat1, treat2, studlab, data = d1, subset=NULL,

sm = "MD", level=0.95, level.comb=0.95,

comb.random=TRUE, reference.group="",

all.treatments=NULL, seq=NULL, tau.preset=NULL,

tol.multiarm = 0.05, warn=TRUE)

}

else if (input$modelCont=="RE" & input$outcomeCont=="SMD"){

netStatic <- netmeta(TE, seTE, treat1, treat2, studlab, data = d1, subset=NULL,

sm = "SMD", level=0.95, level.comb=0.95,

comb.random=TRUE, reference.group="",

all.treatments=NULL, seq=NULL, tau.preset=NULL,

tol.multiarm = 0.05, warn=TRUE)

}

else if (input$modelCont=="FE" & input$outcomeCont=="MD"){

netStatic <- netmeta(TE, seTE, treat1, treat2, studlab, data = d1, subset=NULL,

sm = "MD", level=0.95, level.comb=0.95,

comb.fixed=TRUE, reference.group="",

all.treatments=NULL, seq=NULL, tau.preset=NULL,

tol.multiarm = 0.05, warn=TRUE)

}

else {

netStatic <- netmeta(TE, seTE, treat1, treat2, studlab, data = d1, subset=NULL,

sm = "SMD", level=0.95, level.comb=0.95,

comb.fixed=TRUE, reference.group="",

all.treatments=NULL, seq=NULL, tau.preset=NULL,

tol.multiarm = 0.05, warn=TRUE)

}

if (input$modelCont=="RE" & input$rankoptsCont=="good"){

league <- netleague(netStatic, comb.random=TRUE, digits =2, seq= netrank(netStatic, small = "good"))

leaguedf<- as.data.frame(league$random)

}

else if (input$modelCont=="FE" & input$rankoptsCont=="good"){

league <- netleague(netStatic, comb.random=FALSE, digits =2, seq= netrank(netStatic, small = "good"))

leaguedf<- as.data.frame(league$fixed)

}

else if (input$modelCont=="RE" & input$rankoptsCont=="bad"){

league <- netleague(netStatic, comb.random=TRUE, digits =2, seq= netrank(netStatic, small = "bad"))

leaguedf<- as.data.frame(league$random)

}

else {

league <- netleague(netStatic, comb.random=FALSE, digits =2, seq= netrank(netStatic, small = "bad"))

leaguedf<- as.data.frame(league$fixed)

}

leaguedf

}

output$rankChartStaticCont<- renderTable(colnames=FALSE,{

withProgress(message = 'Loading', value = 0, {

# Number of times we'll go through the loop

n <- 10

for (i in 1:n) {

# Increment the progress bar, and update the detail text.

incProgress(1/n, detail = paste(""))

}

})

make_netrankCont()

})

output$downloadRankCont <- downloadHandler(

filename = function() {

paste('Rank.', '.csv', sep='')

},

content = function(file) {

write.csv({make_netrankCont()}, file)

}

)

##########

### Comparison and rank table updating

##########

make_netrankUpdateCont = function(){

#### same code for inputting the data

newData <- data()

newData1 <- as.data.frame(newData)

if (ncol(newData1)==6){

newData2<-newData1[order(newData1$StudyID, -newData1$T), ]

# create counting variable for number of arms within each study.

newData2$number<- ave(as.numeric(newData2$StudyID),newData2$StudyID,FUN=seq_along)

# reshape

data_wide <- reshape(newData2, timevar = "number",idvar = c("Study", "StudyID"), direction = "wide")

numbertreat=max(newData2$number)

}

else {

data_wide<- newData1

numbertreat=(ncol(newData1)-2)/4

}

# generate additional columns if less than 6 arms.

if (numbertreat < 6) {

for (k in (numbertreat+1):6) {

data_wide[c(paste0("T.",k),paste0("N.",k),paste0("Mean.",k),paste0("SD.",k))]<-NA

}

}

#####

d1<- pairwise(treat=list(T.1,T.2,T.3,T.4,T.5,T.6),n=list(N.1,N.2,N.3,N.4,N.5,N.6),mean=list(Mean.1,Mean.2,Mean.3,Mean.4,Mean.5,Mean.6),sd=list(SD.1,SD.2,SD.3,SD.4,SD.5,SD.6),data=data_wide,sm="MD")

treat_list <- read.csv(text=input$listCont2, sep = "\t")

lstx <- treat_list$Label

ntx <- length(lstx)

# Get subset of data to use

data_sub <- filter(data_wide, !Study %in% input$exclusionCont2)

if (input$outcomeCont=="MD"){

d1_sub<- pairwise(treat=list(T.1,T.2,T.3,T.4,T.5,T.6),n=list(N.1,N.2,N.3,N.4,N.5,N.6),mean=list(Mean.1,Mean.2,Mean.3,Mean.4,Mean.5,Mean.6),sd=list(SD.1,SD.2,SD.3,SD.4,SD.5,SD.6),data=data_sub,sm="MD")

}

else{

d1_sub<- pairwise(treat=list(T.1,T.2,T.3,T.4,T.5,T.6),n=list(N.1,N.2,N.3,N.4,N.5,N.6),mean=list(Mean.1,Mean.2,Mean.3,Mean.4,Mean.5,Mean.6),sd=list(SD.1,SD.2,SD.3,SD.4,SD.5,SD.6),data=data_sub,sm="SMD")

}

d1_sub$treat1 <- factor(d1_sub$treat1,

levels = c(1:ntx),

labels = as.character(treat_list$Label))

d1_sub$treat2 <- factor(d1_sub$treat2,

levels = c(1:ntx),

labels = as.character(treat_list$Label))

# Network meta-analysis

if (input$modelCont=="RE" & input$outcomeCont=="MD"){

net1 <- netmeta(TE, seTE, treat1, treat2, studlab, data = d1_sub, subset=NULL,

sm = "MD", level=0.95, level.comb=0.95,

comb.random=TRUE, reference.group="",

all.treatments=NULL, seq=NULL, tau.preset=NULL,

tol.multiarm = 0.05, warn=TRUE)

}

else if (input$modelCont=="RE" & input$outcomeCont=="SMD"){

net1 <- netmeta(TE, seTE, treat1, treat2, studlab, data = d1_sub, subset=NULL,

sm = "SMD", level=0.95, level.comb=0.95,

comb.random=TRUE, reference.group="",

all.treatments=NULL, seq=NULL, tau.preset=NULL,

tol.multiarm = 0.05, warn=TRUE)

}

else if (input$modelCont=="FE" & input$outcomeCont=="MD"){

net1 <- netmeta(TE, seTE, treat1, treat2, studlab, data = d1_sub, subset=NULL,

sm = "MD", level=0.95, level.comb=0.95,

comb.fixed=TRUE, reference.group="",

all.treatments=NULL, seq=NULL, tau.preset=NULL,

tol.multiarm = 0.05, warn=TRUE)

}

else {

net1 <- netmeta(TE, seTE, treat1, treat2, studlab, data = d1_sub, subset=NULL,

sm = "SMD", level=0.95, level.comb=0.95,

comb.fixed=TRUE, reference.group="",

all.treatments=NULL, seq=NULL, tau.preset=NULL,

tol.multiarm = 0.05, warn=TRUE)

}

if (input$modelCont=="RE" & input$rankoptsCont=="good"){

league1 <- netleague(net1, comb.random=TRUE, digits =2, seq= netrank(net1, small = "good"))

leaguedf1<- as.data.frame(league1$random)

}

else if (input$modelCont=="FE" & input$rankoptsCont=="good"){

league1 <- netleague(net1, comb.random=FALSE, digits =2, seq= netrank(net1, small = "good"))

leaguedf1<- as.data.frame(league1$fixed)

}

else if (input$modelCont=="RE" & input$rankoptsCont=="bad"){

league1 <- netleague(net1, comb.random=TRUE, digits =2, seq= netrank(net1, small = "bad"))

leaguedf1<- as.data.frame(league1$random)

}

else {

league1 <- netleague(net1, comb.random=FALSE, digits =2, seq= netrank(net1, small = "bad"))

leaguedf1<- as.data.frame(league1$fixed)

}

leaguedf1

}

output$rankChartUpdatingCont<- renderTable(colnames=FALSE,{

withProgress(message = 'Loading', value = 0, {

# Number of times we'll go through the loop

n <- 10

for (i in 1:n) {

# Increment the progress bar, and update the detail text.

incProgress(1/n, detail = paste(""))

}

})

make_netrankUpdateCont()

})

output$downloadRankUpdateCont <- downloadHandler(

filename = function() {

paste('RankUpdate.', '.csv', sep='')

},

content = function(file) {

write.csv({make_netrankUpdateCont()}, file)

}

)

################

### Forest Plot - Updating ##

################

make_netCompCont = function(){

#### same code for inputting the data

newData <- data()

newData1 <- as.data.frame(newData)

if (ncol(newData1)==6){

newData2<-newData1[order(newData1$StudyID, -newData1$T), ]

# create counting variable for number of arms within each study.

newData2$number<- ave(as.numeric(newData2$StudyID),newData2$StudyID,FUN=seq_along)

# reshape

data_wide <- reshape(newData2, timevar = "number",idvar = c("Study", "StudyID"), direction = "wide")

numbertreat=max(newData2$number)

}

else {

data_wide<- newData1

numbertreat=(ncol(newData1)-2)/4

}

# generate additional columns if less than 6 arms.

if (numbertreat < 6) {

for (k in (numbertreat+1):6) {

data_wide[c(paste0("T.",k),paste0("N.",k),paste0("Mean.",k),paste0("SD.",k))]<-NA

}

}

#####

treat_list <- read.csv(text=input$listCont2, sep = "\t")

lstx <- treat_list$Label

ntx <- length(lstx)

# Get subset of data to use

data_sub <- filter(data_wide, !Study %in% input$exclusionCont2)

if (input$outcomeCont=="MD"){

d1_sub<- pairwise(treat=list(T.1,T.2,T.3,T.4,T.5,T.6),n=list(N.1,N.2,N.3,N.4,N.5,N.6),mean=list(Mean.1,Mean.2,Mean.3,Mean.4,Mean.5,Mean.6),sd=list(SD.1,SD.2,SD.3,SD.4,SD.5,SD.6),data=data_sub,sm="MD")

}

else{

d1_sub<- pairwise(treat=list(T.1,T.2,T.3,T.4,T.5,T.6),n=list(N.1,N.2,N.3,N.4,N.5,N.6),mean=list(Mean.1,Mean.2,Mean.3,Mean.4,Mean.5,Mean.6),sd=list(SD.1,SD.2,SD.3,SD.4,SD.5,SD.6),data=data_sub,sm="SMD")

}

d1_sub$treat1 <- factor(d1_sub$treat1,

levels = c(1:ntx),

labels = as.character(treat_list$Label))

d1_sub$treat2 <- factor(d1_sub$treat2,

levels = c(1:ntx),

labels = as.character(treat_list$Label))

# Network meta-analysis for subgroup

if (input$modelCont=="RE" & input$outcomeCont=="MD"){

net1 <- netmeta(TE, seTE, treat1, treat2, studlab, data = d1_sub, subset=NULL,

sm = "MD", level=0.95, level.comb=0.95,

comb.random=TRUE, reference.group =as.character(lstx[1]),

all.treatments=NULL, seq=NULL, tau.preset=NULL,

tol.multiarm = 0.05, warn=TRUE)

forest(net1, reference.group=as.character(lstx[1]), pooled="random")

}

else if (input$modelCont=="RE" & input$outcomeCont=="SMD"){

net1 <- netmeta(TE, seTE, treat1, treat2, studlab, data = d1_sub, subset=NULL,

sm = "SMD", level=0.95, level.comb=0.95,

comb.random=TRUE, reference.group =as.character(lstx[1]),

all.treatments=NULL, seq=NULL, tau.preset=NULL,

tol.multiarm = 0.05, warn=TRUE)

forest(net1, reference.group=as.character(lstx[1]), pooled="random")

}

else if (input$modelCont=="FE" & input$outcomeCont=="MD"){

net1 <- netmeta(TE, seTE, treat1, treat2, studlab, data = d1_sub, subset=NULL,

sm = "MD", level=0.95, level.comb=0.95,

comb.fixed=TRUE, reference.group =as.character(lstx[1]),

all.treatments=NULL, seq=NULL, tau.preset=NULL,

tol.multiarm = 0.05, warn=TRUE)

forest(net1, reference.group=as.character(lstx[1]), pooled="fixed")

}

else {

net1 <- netmeta(TE, seTE, treat1, treat2, studlab, data = d1_sub, subset=NULL,

sm = "SMD", level=0.95, level.comb=0.95,

comb.fixed=TRUE, reference.group =as.character(lstx[1]),

all.treatments=NULL, seq=NULL, tau.preset=NULL,

tol.multiarm = 0.05, warn=TRUE)

forest(net1, reference.group=as.character(lstx[1]), pooled="fixed")

}

output$refcont3<- renderText({"All outcomes are versus the reference treatment (treatment labelled 1)"})

}

output$SFPUpdatingContComp <- renderPlot({

withProgress(message = 'Loading', value = 0, {

# Number of times we'll go through the loop

n <- 10

for (i in 1:n) {

# Increment the progress bar, and update the detail text.

incProgress(1/n, detail = paste(""))

}

})

make_netCompCont()

title("Results with studies excluded")

})

output$downloadCompCont<- downloadHandler(

filename = function() {

paste0('Excluded_studies.', input$format_freq4)

},

content = function(file) {

if (input$format_freq4=="PDF"){pdf(file=file)}

else {png(file=file)}

make_netCompCont()

dev.off()

}

)

###### text under the forest plot - updating

textunder=function() {

#### same code for inputting the data

newData <- data()

newData1 <- as.data.frame(newData)

if (ncol(newData1)==6){

newData2<-newData1[order(newData1$StudyID, -newData1$T), ]

# create counting variable for number of arms within each study.

newData2$number<- ave(as.numeric(newData2$StudyID),newData2$StudyID,FUN=seq_along)

# reshape

data_wide <- reshape(newData2, timevar = "number",idvar = c("Study", "StudyID"), direction = "wide")

numbertreat=max(newData2$number)

}

else {

data_wide<- newData1

numbertreat=(ncol(newData1)-2)/4

}

# generate additional columns if less than 6 arms.

if (numbertreat < 6) {

for (k in (numbertreat+1):6) {

data_wide[c(paste0("T.",k),paste0("N.",k),paste0("Mean.",k),paste0("SD.",k))]<-NA

}

}

#####

treat_list <- read.csv(text=input$listCont2, sep = "\t")

lstx <- treat_list$Label

ntx <- length(lstx)

# Get subset of data to use

data_sub <- filter(data_wide, !Study %in% input$exclusionCont2)

if (input$outcomeCont=="MD"){

d1_sub<- pairwise(treat=list(T.1,T.2,T.3,T.4,T.5,T.6),n=list(N.1,N.2,N.3,N.4,N.5,N.6),mean=list(Mean.1,Mean.2,Mean.3,Mean.4,Mean.5,Mean.6),sd=list(SD.1,SD.2,SD.3,SD.4,SD.5,SD.6),data=data_sub,sm="MD")

}

else{

d1_sub<- pairwise(treat=list(T.1,T.2,T.3,T.4,T.5,T.6),n=list(N.1,N.2,N.3,N.4,N.5,N.6),mean=list(Mean.1,Mean.2,Mean.3,Mean.4,Mean.5,Mean.6),sd=list(SD.1,SD.2,SD.3,SD.4,SD.5,SD.6),data=data_sub,sm="SMD")

}

d1_sub$treat1 <- factor(d1_sub$treat1,

levels = c(1:ntx),

labels = as.character(treat_list$Label))

d1_sub$treat2 <- factor(d1_sub$treat2,

levels = c(1:ntx),

labels = as.character(treat_list$Label))

# Network meta-analysis

if (input$modelCont=="RE" & input$outcomeCont=="MD"){

net1 <- netmeta(TE, seTE, treat1, treat2, studlab, data = d1_sub, subset=NULL,

sm = "MD", level=0.95, level.comb=0.95,

comb.random=TRUE, reference.group =as.character(lstx[1]),

all.treatments=NULL, seq=NULL, tau.preset=NULL,

tol.multiarm = 0.05, warn=TRUE)

}

else if (input$modelCont=="RE" & input$outcomeCont=="SMD"){

net1 <- netmeta(TE, seTE, treat1, treat2, studlab, data = d1_sub, subset=NULL,

sm = "SMD", level=0.95, level.comb=0.95,

comb.random=TRUE, reference.group =as.character(lstx[1]),

all.treatments=NULL, seq=NULL, tau.preset=NULL,

tol.multiarm = 0.05, warn=TRUE)

}

else if (input$modelCont=="FE" & input$outcomeCont=="MD"){

net1 <- netmeta(TE, seTE, treat1, treat2, studlab, data = d1_sub, subset=NULL,

sm = "MD", level=0.95, level.comb=0.95,

comb.fixed=TRUE, reference.group =as.character(lstx[1]),

all.treatments=NULL, seq=NULL, tau.preset=NULL,

tol.multiarm = 0.05, warn=TRUE)

}

else {

net1 <- netmeta(TE, seTE, treat1, treat2, studlab, data = d1_sub, subset=NULL,

sm = "SMD", level=0.95, level.comb=0.95,

comb.fixed=TRUE, reference.group =as.character(lstx[1]),

all.treatments=NULL, seq=NULL, tau.preset=NULL,

tol.multiarm = 0.05, warn=TRUE)

}

tau<- round(net1$tau,2)

list(tau=tau, net1_k=net1$k, net1_n=net1$n )

}

output$textcont5 <-renderText({

textunder_list=textunder()

tau=textunder_list$tau

net1_k=textunder_list$net1_k

net1_n=textunder_list$net1_n

if (input$modelCont=="RE"){

paste("Between-study standard deviation:", tau,

", Number of studies:", net1_k,

", Number of treatments:", net1_n)}

else{paste("Between-study standard deviation set at 0. Number of studies:", net1_k,

", Number of treatments:", net1_n)}

}

)

##########

### Forest Plot - static

##########

make_netCompCont2 = function() {

#### same code for inputting the data

newData <- data()

newData1 <- as.data.frame(newData)

if (ncol(newData1)==6){

newData2<-newData1[order(newData1$StudyID, -newData1$T), ]

# create counting variable for number of arms within each study.

newData2$number<- ave(as.numeric(newData2$StudyID),newData2$StudyID,FUN=seq_along)

# reshape

data_wide <- reshape(newData2, timevar = "number",idvar = c("Study", "StudyID"), direction = "wide")

numbertreat=max(newData2$number)

}

else {

data_wide<- newData1

numbertreat=(ncol(newData1)-2)/4

}

# generate additional columns if less than 6 arms.

if (numbertreat < 6) {

for (k in (numbertreat+1):6) {

data_wide[c(paste0("T.",k),paste0("N.",k),paste0("Mean.",k),paste0("SD.",k))]<-NA

}

}

#####

if (input$outcomeCont=="MD"){

d1<- pairwise(treat=list(T.1,T.2,T.3,T.4,T.5,T.6),n=list(N.1,N.2,N.3,N.4,N.5,N.6),mean=list(Mean.1,Mean.2,Mean.3,Mean.4,Mean.5,Mean.6),sd=list(SD.1,SD.2,SD.3,SD.4,SD.5,SD.6),data=data_wide,sm="MD")

}

else{

d1<- pairwise(treat=list(T.1,T.2,T.3,T.4,T.5,T.6),n=list(N.1,N.2,N.3,N.4,N.5,N.6),mean=list(Mean.1,Mean.2,Mean.3,Mean.4,Mean.5,Mean.6),sd=list(SD.1,SD.2,SD.3,SD.4,SD.5,SD.6),data=data_wide,sm="SMD")

}

treat_list <- read.csv(text=input$listCont2, sep = "\t")

lstx <- treat_list$Label

ntx <- length(lstx)

d1$treat1 <- factor(d1$treat1,

levels = c(1:ntx),

labels = as.character(treat_list$Label))

d1$treat2 <- factor(d1$treat2,

levels = c(1:ntx),

labels = as.character(treat_list$Label))

# Static NMA of all studies

if (input$modelCont=="RE" & input$outcomeCont=="MD"){

netStatic <- netmeta(TE, seTE, treat1, treat2, studlab, data = d1, subset=NULL,

sm = "MD", level=0.95, level.comb=0.95,

comb.random=TRUE, reference.group=as.character(lstx[1]),

all.treatments=NULL, seq=NULL, tau.preset=NULL,

tol.multiarm = 0.05, warn=TRUE)

forest(netStatic, reference.group=as.character(lstx[1]), pooled="random")

}

else if (input$modelCont=="RE" & input$outcomeCont=="SMD"){

netStatic <- netmeta(TE, seTE, treat1, treat2, studlab, data = d1, subset=NULL,

sm = "SMD", level=0.95, level.comb=0.95,

comb.random=TRUE, reference.group=as.character(lstx[1]),

all.treatments=NULL, seq=NULL, tau.preset=NULL,

tol.multiarm = 0.05, warn=TRUE)

forest(netStatic, reference.group=as.character(lstx[1]), pooled="random")

}

else if (input$modelCont=="FE" & input$outcomeCont=="MD"){

netStatic <- netmeta(TE, seTE, treat1, treat2, studlab, data = d1, subset=NULL,

sm = "MD", level=0.95, level.comb=0.95,

comb.fixed=TRUE, reference.group =as.character(lstx[1]),

all.treatments=NULL, seq=NULL, tau.preset=NULL,

tol.multiarm = 0.05, warn=TRUE)

forest(netStatic, reference.group=as.character(lstx[1]), pooled="fixed")

}

else {

netStatic <- netmeta(TE, seTE, treat1, treat2, studlab, data = d1, subset=NULL,

sm = "SMD", level=0.95, level.comb=0.95,

comb.fixed=TRUE, reference.group =as.character(lstx[1]),

all.treatments=NULL, seq=NULL, tau.preset=NULL,

tol.multiarm = 0.05, warn=TRUE)

forest(netStatic, reference.group=as.character(lstx[1]), pooled="fixed")

}

output$refcont4<- renderText({"All outcomes are versus the reference treatment (treatment labelled 1)"})

}

output$ComparisonCont2<- renderPlot({

make_netCompCont2()

title("Results for all studies")

})

######## Forest plot - text under the plot

output$textcompcont<- renderText({

#### same code for inputting the data

newData <- data()

newData1 <- as.data.frame(newData)

if (ncol(newData1)==6){

newData2<-newData1[order(newData1$StudyID, -newData1$T), ]

# create counting variable for number of arms within each study.

newData2$number<- ave(as.numeric(newData2$StudyID),newData2$StudyID,FUN=seq_along)

# reshape

data_wide <- reshape(newData2, timevar = "number",idvar = c("Study", "StudyID"), direction = "wide")

numbertreat=max(newData2$number)

}

else {

data_wide<- newData1

numbertreat=(ncol(newData1)-2)/4

}

# generate additional columns if less than 6 arms.

if (numbertreat < 6) {

for (k in (numbertreat+1):6) {

data_wide[c(paste0("T.",k),paste0("N.",k),paste0("Mean.",k),paste0("SD.",k))]<-NA

}

}

#####

if (input$outcomeCont=="MD"){

d1<- pairwise(treat=list(T.1,T.2,T.3,T.4,T.5,T.6),n=list(N.1,N.2,N.3,N.4,N.5,N.6),mean=list(Mean.1,Mean.2,Mean.3,Mean.4,Mean.5,Mean.6),sd=list(SD.1,SD.2,SD.3,SD.4,SD.5,SD.6),data=data_wide,sm="MD")

}

else{

d1<- pairwise(treat=list(T.1,T.2,T.3,T.4,T.5,T.6),n=list(N.1,N.2,N.3,N.4,N.5,N.6),mean=list(Mean.1,Mean.2,Mean.3,Mean.4,Mean.5,Mean.6),sd=list(SD.1,SD.2,SD.3,SD.4,SD.5,SD.6),data=data_wide,sm="SMD")

}

treat_list <- read.csv(text=input$listCont2, sep = "\t")

lstx <- treat_list$Label

ntx <- length(lstx)

d1$treat1 <- factor(d1$treat1,

levels = c(1:ntx),

labels = as.character(treat_list$Label))

d1$treat2 <- factor(d1$treat2,

levels = c(1:ntx),

labels = as.character(treat_list$Label))

# Static NMA of all studies

if (input$modelCont=="RE" & input$outcomeCont=="MD"){

netStatic <- netmeta(TE, seTE, treat1, treat2, studlab, data = d1, subset=NULL,

sm = "MD", level=0.95, level.comb=0.95,

comb.random=TRUE, reference.group="",

all.treatments=NULL, seq=NULL, tau.preset=NULL,

tol.multiarm = 0.05, warn=TRUE)

}

else if (input$modelCont=="RE" & input$outcomeCont=="SMD"){

netStatic <- netmeta(TE, seTE, treat1, treat2, studlab, data = d1, subset=NULL,

sm = "SMD", level=0.95, level.comb=0.95,

comb.random=TRUE, reference.group="",

all.treatments=NULL, seq=NULL, tau.preset=NULL,

tol.multiarm = 0.05, warn=TRUE)

}

else if (input$modelCont=="FE" & input$outcomeCont=="MD"){

netStatic <- netmeta(TE, seTE, treat1, treat2, studlab, data = d1, subset=NULL,

sm = "MD", level=0.95, level.comb=0.95,

comb.fixed=TRUE, reference.group="",

all.treatments=NULL, seq=NULL, tau.preset=NULL,

tol.multiarm = 0.05, warn=TRUE)

}

else {

netStatic <- netmeta(TE, seTE, treat1, treat2, studlab, data = d1, subset=NULL,

sm = "SMD", level=0.95, level.comb=0.95,

comb.fixed=TRUE, reference.group="",

all.treatments=NULL, seq=NULL, tau.preset=NULL,

tol.multiarm = 0.05, warn=TRUE)

}

tau<- round(netStatic$tau,2)

if (input$modelCont=="RE"){

paste("Between-study standard deviation:", tau,

", Number of studies:", netStatic$k,

", Number of treatments:", netStatic$n)}

else{paste("Between-study standard deviation set at 0. Number of studies:", netStatic$k,

", Number of treatments:", netStatic$n)}

})

output$downloadCompCont2 <- downloadHandler(

filename = function() {

paste0('All_studies.', input$format_freq3)

},

content = function(file) {

if (input$format_freq3=="PDF"){pdf(file=file)}

else {png(file=file)}

make_netCompCont2()

dev.off()

}

)

############################

###### Inconsistency - all studies #######

############################

make_InconCont = function() {

#### same code for inputting the data

newData <- data()

newData1 <- as.data.frame(newData)

if (ncol(newData1)==6){

newData2<-newData1[order(newData1$StudyID, -newData1$T), ]

# create counting variable for number of arms within each study.

newData2$number<- ave(as.numeric(newData2$StudyID),newData2$StudyID,FUN=seq_along)

# reshape

data_wide <- reshape(newData2, timevar = "number",idvar = c("Study", "StudyID"), direction = "wide")

numbertreat=max(newData2$number)

}

else {

data_wide<- newData1

numbertreat=(ncol(newData1)-2)/4

}

# generate additional columns if less than 6 arms.

if (numbertreat < 6) {

for (k in (numbertreat+1):6) {

data_wide[c(paste0("T.",k),paste0("N.",k),paste0("Mean.",k),paste0("SD.",k))]<-NA

}

}

#####

if (input$outcomeCont=="MD"){

d1<- pairwise(treat=list(T.1,T.2,T.3,T.4,T.5,T.6),n=list(N.1,N.2,N.3,N.4,N.5,N.6),mean=list(Mean.1,Mean.2,Mean.3,Mean.4,Mean.5,Mean.6),sd=list(SD.1,SD.2,SD.3,SD.4,SD.5,SD.6),data=data_wide,sm="MD")

}

else{

d1<- pairwise(treat=list(T.1,T.2,T.3,T.4,T.5,T.6),n=list(N.1,N.2,N.3,N.4,N.5,N.6),mean=list(Mean.1,Mean.2,Mean.3,Mean.4,Mean.5,Mean.6),sd=list(SD.1,SD.2,SD.3,SD.4,SD.5,SD.6),data=data_wide,sm="SMD")

}

treat_list <- read.csv(text=input$listCont2, sep = "\t")

lstx <- treat_list$Label

ntx <- length(lstx)

d1$treat1 <- factor(d1$treat1,

levels = c(1:ntx),

labels = as.character(treat_list$Label))

d1$treat2 <- factor(d1$treat2,

levels = c(1:ntx),

labels = as.character(treat_list$Label))

# Network meta-analysis

if (input$modelCont=="RE" & input$outcomeCont=="MD"){

net1 <- netmeta(TE, seTE, treat1, treat2, studlab, data = d1, subset=NULL,

sm = "MD", level=0.95, level.comb=0.95,

comb.random=TRUE, reference.group =as.character(lstx[1]),

all.treatments=NULL, seq=NULL, tau.preset=NULL,

tol.multiarm = 0.05, warn=TRUE)

}

else if (input$modelCont=="RE" & input$outcomeCont=="SMD"){

net1 <- netmeta(TE, seTE, treat1, treat2, studlab, data = d1, subset=NULL,

sm = "SMD", level=0.95, level.comb=0.95,

comb.random=TRUE, reference.group =as.character(lstx[1]),

all.treatments=NULL, seq=NULL, tau.preset=NULL,

tol.multiarm = 0.05, warn=TRUE)

}

else if (input$modelCont=="FE" & input$outcomeCont=="MD"){

net1 <- netmeta(TE, seTE, treat1, treat2, studlab, data = d1, subset=NULL,

sm = "MD", level=0.95, level.comb=0.95,

comb.fixed=TRUE, reference.group =as.character(lstx[1]),

all.treatments=NULL, seq=NULL, tau.preset=NULL,

tol.multiarm = 0.05, warn=TRUE)

}

else {

net1 <- netmeta(TE, seTE, treat1, treat2, studlab, data = d1, subset=NULL,

sm = "SMD", level=0.95, level.comb=0.95,

comb.fixed=TRUE, reference.group =as.character(lstx[1]),

all.treatments=NULL, seq=NULL, tau.preset=NULL,

tol.multiarm = 0.05, warn=TRUE)

}

incona<- netsplit(net1)

Comparison<- incona$comparison

No.Studies<- as.integer(incona$k)

if (input$modelCont=="RE"){

Direct<- incona$direct.random$TE

Indirect<- incona$indirect.random$TE

Difference<- incona$compare.random$TE

Diff_95CI_lower<- incona$compare.random$lower

Diff_95CI_upper<- incona$compare.random$upper

NMA<- incona$random$TE

pValue<- incona$compare.random$p}

else{

Direct<- incona$direct.fixed$TE

Indirect<- incona$indirect.fixed$TE

Difference<- incona$compare.fixed$TE

Diff_95CI_lower<- incona$compare.fixed$lower

Diff_95CI_upper<- incona$compare.fixed$upper

NMA<- incona$fixed$TE

pValue<- incona$compare.fixed$p}

df<- data.frame(Comparison, No.Studies, NMA, Direct, Indirect, Difference, Diff_95CI_lower, Diff_95CI_upper, pValue)

}

output$Incon1<- renderTable(colnames=TRUE, {

make_InconCont()}

)

output$downloadInconCont <- downloadHandler(

filename = function() {

paste('Inconsistency.', '.csv', sep='')

},

content = function(file) {

write.csv({make_InconCont()}, file)

}

)

############################

###### Inconsistency - updating #######

############################

make_InconCont_sub = function(){

#### same code for inputting the data

newData <- data()

newData1 <- as.data.frame(newData)

if (ncol(newData1)==6){

newData2<-newData1[order(newData1$StudyID, -newData1$T), ]

# create counting variable for number of arms within each study.

newData2$number<- ave(as.numeric(newData2$StudyID),newData2$StudyID,FUN=seq_along)

# reshape

data_wide <- reshape(newData2, timevar = "number",idvar = c("Study", "StudyID"), direction = "wide")

numbertreat=max(newData2$number)

}

else {

data_wide<- newData1

numbertreat=(ncol(newData1)-2)/4

}

# generate additional columns if less than 6 arms.

if (numbertreat < 6) {

for (k in (numbertreat+1):6) {

data_wide[c(paste0("T.",k),paste0("N.",k),paste0("Mean.",k),paste0("SD.",k))]<-NA

}

}

#####

# Get subset of data to use

data_sub <- filter(data_wide, !Study %in% input$exclusionCont2)

if (input$outcomeCont=="MD"){

d1_sub<- pairwise(treat=list(T.1,T.2,T.3,T.4,T.5,T.6),n=list(N.1,N.2,N.3,N.4,N.5,N.6),mean=list(Mean.1,Mean.2,Mean.3,Mean.4,Mean.5,Mean.6),sd=list(SD.1,SD.2,SD.3,SD.4,SD.5,SD.6),data=data_sub,sm="MD")

}

else{

d1_sub<- pairwise(treat=list(T.1,T.2,T.3,T.4,T.5,T.6),n=list(N.1,N.2,N.3,N.4,N.5,N.6),mean=list(Mean.1,Mean.2,Mean.3,Mean.4,Mean.5,Mean.6),sd=list(SD.1,SD.2,SD.3,SD.4,SD.5,SD.6),data=data_sub,sm="SMD")

}

treat_list <- read.csv(text=input$listCont2, sep = "\t")

lstx <- treat_list$Label

ntx <- length(lstx)

d1_sub$treat1 <- factor(d1_sub$treat1,

levels = c(1:ntx),

labels = as.character(treat_list$Label))

d1_sub$treat2 <- factor(d1_sub$treat2,

levels = c(1:ntx),

labels = as.character(treat_list$Label))

# Network meta-analysis

if (input$modelCont=="RE" & input$outcomeCont=="MD"){

net1 <- netmeta(TE, seTE, treat1, treat2, studlab, data = d1_sub, subset=NULL,

sm = "MD", level=0.95, level.comb=0.95,

comb.random=TRUE, reference.group =as.character(lstx[1]),

all.treatments=NULL, seq=NULL, tau.preset=NULL,

tol.multiarm = 0.05, warn=TRUE)

}

else if (input$modelCont=="RE" & input$outcomeCont=="SMD"){

net1 <- netmeta(TE, seTE, treat1, treat2, studlab, data = d1_sub, subset=NULL,

sm = "SMD", level=0.95, level.comb=0.95,

comb.random=TRUE, reference.group =as.character(lstx[1]),

all.treatments=NULL, seq=NULL, tau.preset=NULL,

tol.multiarm = 0.05, warn=TRUE)

}

else if (input$modelCont=="FE" & input$outcomeCont=="MD"){

net1 <- netmeta(TE, seTE, treat1, treat2, studlab, data = d1_sub, subset=NULL,

sm = "MD", level=0.95, level.comb=0.95,

comb.fixed=TRUE, reference.group =as.character(lstx[1]),

all.treatments=NULL, seq=NULL, tau.preset=NULL,

tol.multiarm = 0.05, warn=TRUE)

}

else {

net1 <- netmeta(TE, seTE, treat1, treat2, studlab, data = d1_sub, subset=NULL,

sm = "SMD", level=0.95, level.comb=0.95,

comb.fixed=TRUE, reference.group =as.character(lstx[1]),

all.treatments=NULL, seq=NULL, tau.preset=NULL,

tol.multiarm = 0.05, warn=TRUE)

}

incona<- netsplit(net1)

Comparison<- incona$comparison

No.Studies<- as.integer(incona$k)

if (input$modelCont=="RE"){

Direct<- incona$direct.random$TE

Indirect<- incona$indirect.random$TE

Difference<- incona$compare.random$TE

Diff_95CI_lower<- incona$compare.random$lower

Diff_95CI_upper<- incona$compare.random$upper

NMA<- incona$random$TE

pValue<- incona$compare.random$p}

else{

Direct<- incona$direct.fixed$TE

Indirect<- incona$indirect.fixed$TE

Difference<- incona$compare.fixed$TE

Diff_95CI_lower<- incona$compare.fixed$lower

Diff_95CI_upper<- incona$compare.fixed$upper

NMA<- incona$fixed$TE

pValue<- incona$compare.fixed$p}

df<- data.frame(Comparison, No.Studies, NMA, Direct, Indirect, Difference, Diff_95CI_lower, Diff_95CI_upper, pValue)

}

output$Incon2<- renderTable(colnames=TRUE, {

make_InconCont_sub()}

)

output$downloadInconCont2 <- downloadHandler(

filename = function() {

paste('Inconsistency_sub.', '.csv', sep='')

},

content = function(file) {

write.csv({make_InconCont_sub()}, file)

}

)

})
